# Antarctic Ardley Island terrace — An ideal place to study the marine to terrestrial succession of microbial communities

**DOI:** 10.3389/fmicb.2023.942428

**Published:** 2023-02-06

**Authors:** Potjanicha Nopnakorn, Yumin Zhang, Lin Yang, Fang Peng

**Affiliations:** China Center for Type Culture Collection (CCTCC), College of Life Sciences, Wuhan University, Wuhan, China

**Keywords:** Ardley Island terrace, soil-borne microbial community, maritime Antarctica, amplicon sequencing, marine to terrestrial succession

## Abstract

The study of chronosequences is an effective tool to study the effects of environmental changes or disturbances on microbial community structures, diversity, and the functional properties of ecosystems. Here, we conduct a chronosequence study on the Ardley Island coastal terrace of the Fildes Peninsula, Maritime Antarctica. The results revealed that prokaryotic microorganism communities changed orderly among the six successional stages. Some marine microbial groups could still be found in near-coastal soils of the late stage (lowest stratum). Animal pathogenic bacteria and stress-resistant microorganisms occurred at the greatest level with the longest succession period. The main driving factors for the succession of bacteria, archaea, and fungi along Ardley Island terrace were found through Adonis analysis (PERMANOVA). During analysis, soil elements Mg, Si, and Na were related to the bacterial and archaeal community structure discrepancies, while Al, Ti, K, and Cl were related to the fungal community structure discrepancies. On the other hand, other environmental factors also play an important role in the succession of microbial communities, which could be different among each microorganism. The succession of bacterial communities is greatly affected by pH and water content; archaeal communities are greatly affected by $NH_{4}^{+}$; fungal communities are affected by nutrients such as $NO_{3}^{-}$. In the analysis of the characteristic microorganisms along terrace, the succession of microorganisms was found to be influenced by complex and comprehensive factors. For instance, environmental instability, relationship with plants and ecological niches, and environmental tolerance. The results found that budding reproduction and/or with filamentous appendages bacteria were enriched in the late stage, which might be connected to its tolerance to rapid changes and barren environments. In addition, the decline in ammonia oxidation capacity of Thaumarchaeota archaeade with succession and the evolution of the fungi-plant relationship throughout classes were revealed. Overall, this research improves the understanding of the effect of the marine–to–terrestrial transition of the Ardley Island terrace on microbial communities. These findings will lay the foundation for more in-depth research regarding microbial adaptations and evolutionary mechanisms throughout the marine–terrestrial transition in the future.

## 1. Introduction

In the past, microbial diversities from various chronosequences were studied using different techniques, such as traditional culture methods, and advanced techniques focusing on genetic, structural, and functional diversity. The study of the marine–terrestrial transition of the microbial community is one of the best ways to study a microbial evolution (Dini-Andreote et al., 2016). However, there are no representable research areas to serve as an ideal place to study the marine–terrestrial transition of microbial communities. The studies on the marine–terrestrial transition of microbial communities are mainly focused on salt marsh chronosequence. The salt marsh ecosystem at the island of Schiermonnikoog (The Netherlands) has been formed through sand accumulation and progressive sedimentation of silt and clay particles, which resulted from cyclic tidal inundation. However, the salinity increased in the late stages, since an accretion and accumulation effect (Dini-Andreote et al., 2014). The salt content in salt marsh chronosequence differs from the decrease of salt content in the Antarctic marine–terrestrial transition. Unlike other ecosystems, the Antarctic environment is harsh, has slow biological growth, and has less human interference.

**Figure 1:**
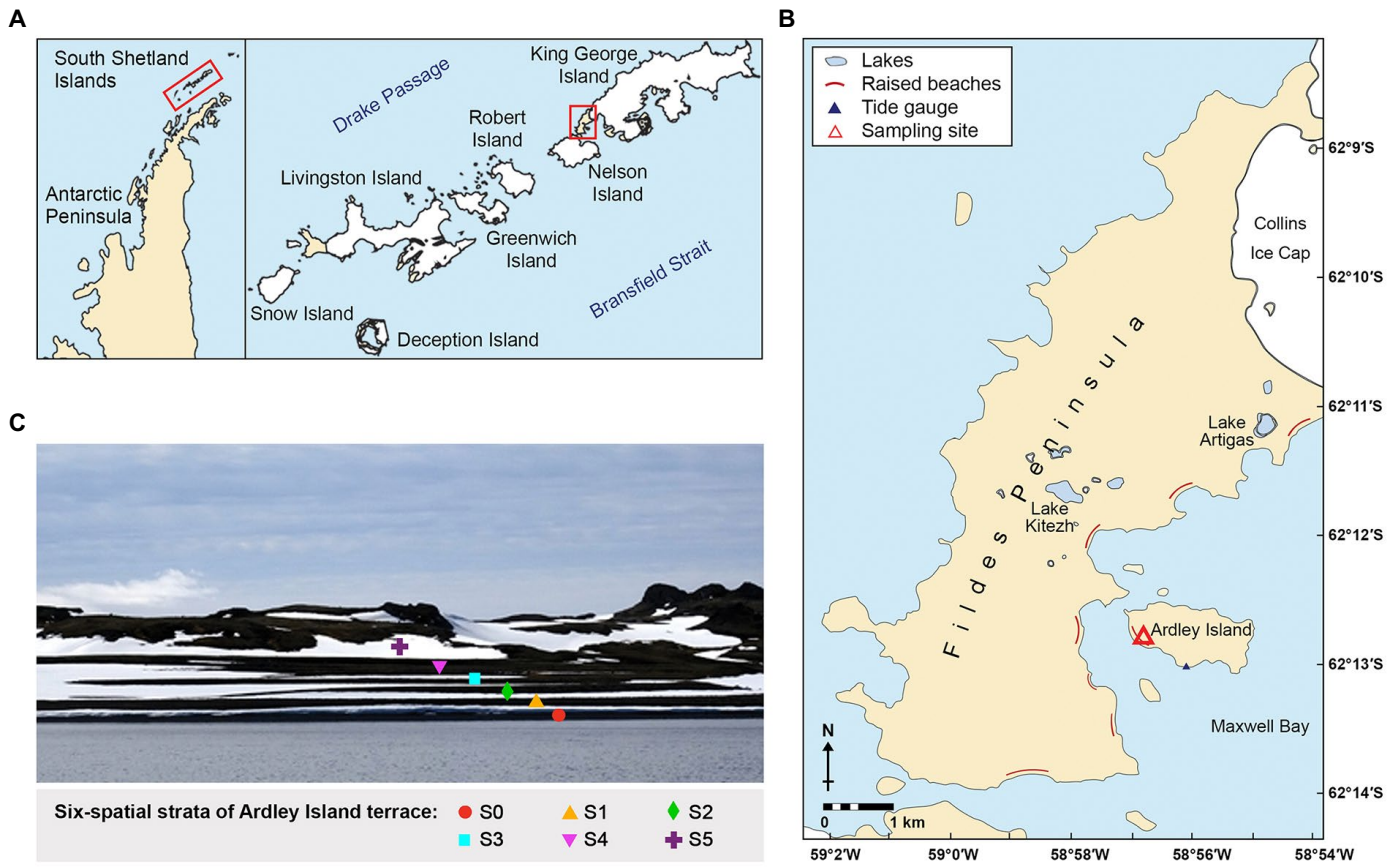
Site location; **(A)** Maps of the Antarctic Peninsula and the South Shetland Islands, **(B)** Locations of the Ardley Island terrace (marked as a red triangle), and **(C)** The Ardley Island terrace: stage S0–S5.

The Fildes Region including the Fildes Peninsula, Ardley Island, and adjacent islands represents one of the largest ice-free areas in the maritime Antarctic. Ardley Island terrace is located on the Fildes Peninsula, King George Island, Antarctica (Figures 1A,B). It has been identified as a raised beach sequence (Palaeobeaches; Boy et al., 2016). The spatial strata of Ardley Island terrace are distinctly separated (Figure 1C). The island rise is relatively low with the highest elevation at 65 m altitude. In geomorphological terms, the area comprises mainly tertiary andesitic-basaltic lavas and tuffs, together with raised beach terraces (Management Plan for Antarctic Specially Protected Area No. 150. 2009). In the middle and late Holocene, Ardley Island was almost completely submerged by seawater due to global warming effects, including melting ice sheet and rising sea levels. At the same time, isostatic uplift due to the melting and weight reduction of the ice sheet has resulted in the formation of chronosequences of raised beaches along with the coastal areas (Ingólfsson et al., 1998; Michel et al., 2014).

In our previous research on the soil microbial community in Fildes Peninsula, Antarctica, we found that the microbial community in this area was affected by geological events. The microorganisms in the coastal uplifted chronosequence were significantly different from those in other areas and were still in an unstable change stage (Zhang et al., 2018). In this area, the coastal uplifted chronosequence of Ardley Island terrace presents a very complete six-spatial strata. The activities of marine animals along with glacial activities and past sea-level changes play a key role in the formation and development of terrace soil during the formation of Ardley Island terrace (Bölter, 2011). Each spatial stage along Ardley Island coastal uplifted chronosequence was dated, and it found that the entire terrace spans an age range of 200-to-7,200-year BP (Boy et al., 2016). Although there is no obvious succession of vegetation in each stage of these terraces, soil development, especially the enrichment of organic matter, is more advanced.

The Ardley Island terrace has less interference from plants, animals, and people. There is no interference from the penguin colonies. The breeding colonies of penguins are located on the eastern side, while the coastal uplifted sequence (Palaeobeaches) is located on the western side of the Ardley Island. Furthermore, because of the clear geological age of the Ardley Island terrace, we chose it as a research site for investigating the microbial communities in the soil along the terrace. We hope to explore the successional pattern of microbial community during marine–to–terrestrial transition with the increase of altitude corresponding to the temporal gradients of coastal uplift, and to lay the foundation to study the evolution of microorganisms during the marine–to–terrestrial transition.

In this study, soil microbial communities of the Ardley Island coastal uplifted chronosequence were studied based on the geological background. The succession of microbial communities in the terraces formed at different time gradients were analyzed. Besides, the change course of microbial communities after marine–to–terrestrial transition, as well as the geological phenomena and evolutionary history that may be reflected by the change of microbial communities were explored.

## 2. Materials and methods

### 2.1. Soil sampling site

The Ardley Island terrace, a typical marine erosion uplift terrace, is located on the Fildes Peninsula, King George Island, Antarctica (Figure 1). The spatial strata of Ardley Island terrace are distinctly separated into 6 successional stages (S0–S5; Table 1). The late stratum, Stage S0 is covered with approximately 95% of vegetation including two to three layers of the lichen *Usnea* sp., a black moss, and encrusted lichens (Boy et al., 2016). The distance from S0 to the coast is approximately 15 meters. Most area of stage S0 was covered by 80% of *Usnea fasciata*. Besides, other vegetations consisted of the green cushion-moss (*Chorisodontium* sp.), the yellow-green bryophyte (*Sanionia uncinata*), the black moss (*Andreaea* sp., *Cladonia Borealis*, *Himantormia lugubris*), and various other encrusted lichens. Stage S1 is covered with 95% of the vegetation. The dominating species, *Usnea fasciata* lichen covers around 70% of the area. Moreover, other vegetations consisting of *Chorisodontium* sp., *Andreaea* sp., *Himantormia lugubris*, *Cornicularia aculeata,* and other encrusted lichens were also detected in this area. Stage S2 is covered by 100% of vegetation with 90% of *Usnea fasciata* as dominating species. Stage S3, S4, and S5 were covered by 100% of vegetation and were comparable regarding vegetation composition to the other stages on the Ardley Island terrace. The organic matter enrichment in stages S4 and S5 is more advanced.

**Table 1:** Local parameters and soil properties of sampled soil sites along the Ardley Island temporal gradient (Boy et al., 2016).

Stage	GPS	Altitude (m)	Est. age (BP)	Soil classification
S0	S62°12′48.9″, W58°56′53.4″	2–3	650–200	Eutric, Arenosol
S1	S62°12′48.0″, W58°56′51.9″	5–6	1,400–650	Dystric, Arenosol
S2	S62°12′47.3″, W58°56′49.6″	6–8	2,200–1,000	Dystric, Arenosol
S3	S62°12′46.4″, W58°56′47.5″	9–16	4,400–2,300	Dystric, Arenosol
S4	S62°12′45.7″, W58°56′46.9″	13–16	5,000	Dystric, Arenosol
S5	S62°12′44.4″, W58°56′42.9″	17–18	7,200	Dystric, Arenosol

Sampling occurred during China’s 33^rd^ Antarctic expedition in January 2017. The GPS coordinates and altitudes are shown in Supplementary S1. The altitude of each spatial stratum (S0–S5) on the Ardley Island terrace is 2.0, 5.0, 6.6, 9.0, 13.0, and 18.0 meters, respectively (Figure 1C). Terrace dating of lichen and shell fossils revealed the ages of the six strata are around 250, 650, 1,000, 2,500, 5,000, and 7,000 years ago (Liu and Cui, 1990; Boy et al., 2016).

A total of 30 soil samples were collected, 10 g at each sampling site. Each stage included five sampling sites with a distance of approximately 0.6–1 km. The distance between the sites was approximately 100–200 m. Each sample had a surface area of 1 × 1 square meter and was collected in triplicate from the A-horizon (10 cm). Soil samples collected for each replicate were taken from five soil cores (5 cm in diameter), mixed thoroughly, then placed in sterile plastic bags. Soil DNA was extracted within 2 h in the laboratory of the Great Wall Station. The remaining soil samples were dried naturally, ground, and passed through 100-mesh sieves for the subsequent determination of soil parameters.

### 2.2. Determination of soil elemental compositions by X-ray fluorescence spectrometry

Soil samples were dried at 105°C for 6 h, then ground to powder. The soil powder was pressed in a 45 mm diameter bore steel die under an approximately 20 t hydraulic press. Each soil sample was formed into a stable soil pie (45 mm diameter, 10 mm height), and then analyzed within a few hours. The soil elemental compositions were determined using X-ray fluorescence spectrometry (Bruker AXS, Germany) with a standardless quantitative analysis method (Handley et al., 2010).

### 2.3. Soil physicochemical parameters measurements

To measure soil pH, 5 g of each soil sample was suspended in 10 ml of deionized water. The pH of each soil suspension was measured using a pH meter (Mettler-Toledo, Switzerland). The Direct Gravimetric method was used to determine the soil moisture content. The weight loss of each soil sample was calculated after drying the soil sample at 105°C until it reached a constant weight. Total organic carbon (TOC) was determined using a TOC analyzer (Vario TOC, Elementar, Germany). Soil $NH_{4}^{+}$ and $NO_{3}^{-}$ content was measured by extraction of 10 g of soil sample with 50 ml of KCl (2 mol/l) solution at 25°C for 1 h. The soil solution mixture was then centrifuged at 3000 g for 5 min. After centrifugation, the clear supernatant was passed through a 0.45-μm filter (Millipore, type GP). Subsequently, the filtrate was analyzed by a continuous flowing analyzer (FIAstar™ 5,000 Analyzer, Foss, Denmark).

### 2.4. Soil DNA extraction, PCR, and Illumina Miseq high-throughput sequencing

Total soil DNA was extracted within 2 h in the laboratory of the Great Wall Station using the DNeasy PowerSoil DNA Isolation Kit (Mo Bio Laboratories, Carlsbad, CA, United States) following the manufacturer’s instructions. DNA concentrations were further measured with a UV spectrophotometer (Eppendorf, Bio Photometer). DNA molecular sizes were estimated by 0.8% agarose gel electrophoresis. The DNA extracts were stored at -20°C for the following PCR amplification. The Illumina Miseq sequencing and sequencing data treatment are described in Supplementary Table S1.

### 2.5. Statistical analyses

Before analysis, the raw sequencing data were demultiplexed and processed using the Quantitative Insights Into Microbial Ecology (QIIME) v. 1.8.0. (Boulder, CO, United States) to remove the low-quality short length reads (< 150 BP), the long polymer sequence (> 8 BP), and the low-quality base sequence.

To obtain high-quality and clean reads, chimeric sequences were identified using USEARCH v. 5.2.236^1^ and then removed. The quality reads were binned into operational taxonomic units (OTUs) at 97% sequence similarity using UCLUST, followed by the selection of a representative sequence for each OTU. The OTUs with a sequence number less than 0.001% of the total sequence number were eliminated (Bokulich et al., 2013). The representative sequence for each OTU was aligned to bacterial and archaea taxa based on the SILVA and Greengenes ribosomal RNA database, and to fungal taxa based on UNITE database (DeSantis et al., 2006; Pruesse et al., 2007; Quast et al., 2013).

To evaluate the alpha diversity of bacteria, archaea, and fungi, operational taxonomic unit (OTU) analyses were carried out with the Sobs and Shannon indices. The analyses were accomplished by using the Mothur v. 1.30.2 software package (Schloss et al., 2009). To analyze the relationships between soil elemental compositions and environmental attributes in the soil samples, principal component analysis (PCA) was performed using R v. 3.3.1. statistical software. In addition, the one-way analysis of variance (ANOVA) method was used in R v. 3.3.1. statistical software to test the significant differences among environmental factors. The ANalysis Of SIMilarities (ANOSIM; Clarke, 1993) as well as the non-parametric Adonis test (Anderson, 2001) with 999 permutations were conducted to compare the differential of the microbial communities in different strata. The Bray-Curtis distance was used to obtain the dissimilarity matrices in the permutational multivariate analysis of variance (PERMANOVA) test for microbial OTU data, which had been normalized by dividing the reads per OTU in a sample by the sum of usable reads in that sample (relative abundances), where an OTU absent from a sample was coded as state 0. In the analysis of the microbial community in the terrace of the Fildes Peninsula, the method of distance-based redundancy analysis (db-RDA) was used to rank the microbial communities based on soil parameters, and the Bray-Curtis distance between communities was analyzed in R v. 3.3.1. statistical software. In addition, the Linear Discriminant Analysis (LDA) Effect Size (LEfSe) was used to identify the taxonomic biomarkers identified in each stratum of the Ardley Island terrace using relative abundances (Segata et al., 2011; Paulson et al., 2013). In the analysis, an alpha parameter significance threshold for the Kruskal-Wallis (KW) test among classes was set to 0.05. The threshold on the logarithmic score of LDA analysis was set to 3.0. The analysis was processed with the Galaxy platform developed by Harvard University (Afgan et al., 2018). Prediction of high-level bacterial phenotypic traits were carried out through BugBase^2^ (Ward et al., 2017) using the Greengenes annotated biom files. The prediction of ecologically relevant functions of microbial taxa was carried out *via* a promising tool, FAPROTAX (Louca et al., 2016).

## 3. Results

### 3.1. Soil elemental compositions and physicochemical properties

**Figure 2:**
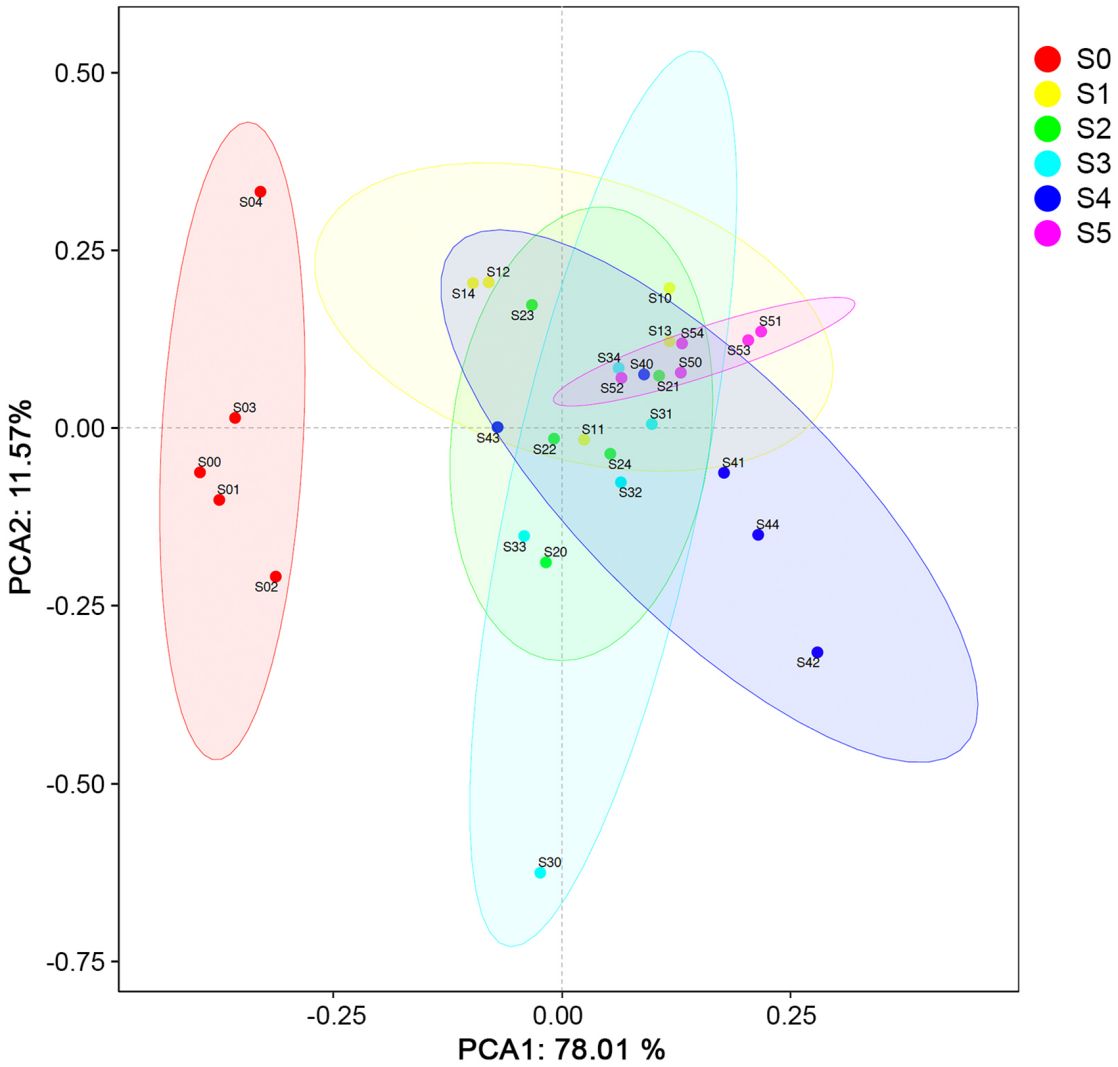
Principal component analysis (PCA) of the normalized soil elemental compositions and physicochemical properties.

A total of 25 elements in the soil samples were quantified by X-ray fluorescence spectrometry. After removing the elements C, N, O, and those elements that only appear in one sample and the abundance is less than 0.01%, the final element composition for data analysis is shown in Supplementary Table S1. The most abundant elements along chronosequence are Si (11.2–27.1%), Al (6.1–12.0%), Fe (5.6–8.0%), Ca (1.6–5.8%), Na (1.4–2.8%), and Mg (0.7–2.7%). Compared with other strata, six elements including Al (9.3–9.8%), K (1.1–1.2%), Mg (2.1–2.2%), Mn (0.1%), Na (2.6–2.8%) and Si (25.5–27.1%) were more abundant in S0 stratum, which made it different from other strata in the Ardley Island terrace (Figure 2).

The physiochemical properties of soil samples along the Ardley Island terrace are shown in Table 2. The ANOVA analysis revealed a highly significant difference in soil moisture contents and pH between the last two successional stages (stage S4 and S5) and the late stages (stage S0, S1, S2, and S3). The S0 stage revealed lower moisture contents, total organic carbon (TOC), and soil $NH_{4}^{+}$ than in the other stages. On the other hand, soil pH values were high in stage S0, and lower in the other stages.

**Table 2:** Soil physiochemical properties investigated in this study.

	Moisture contents (%)	pH	TOC (%)	$NO_{3}^{-}$(mg/kg)	$NH_{4}^{+}$(mg/kg)
S0	14.8^b^ (4.9)	6.7^a^ (0.1)	2.3^b^ (2.32)	2.1^a, b^ (2.4)	7.0^b^ (1.4)
S1	32.1^a^ (6.0)	5.9^b^ (0.5)	10.14^a, b^ (3.17)	2.5^a, b^ (1.6)	29.2^a^ (9.6)
S2	37.9^a^ (6.4)	5.9^b^ (0.1)	7.24^b^ (2.78)	1.9^b^ (0.5)	29.5^a^ (6.1)
S3	35.1^a^ (7.2)	5.6^b^ (0.5)	6.12^b^ (3.82)	3.2^a^ (0.6)	32.9^a^ (5.2)
S4	39.3^a^ (9.1)	5.9^b^ (0.2)	8.48^a, b^ (1.64)	3.7^a^ (0.9)	48.4^a^ (20.8)
S5	41.6^a^ (4.5)	5.8^b^ (0.2)	12.82^a^ (2.30)	1.8^b^ (0.9)	40.7^a^ (6.8)

The values with different superscripts indicate that the index has a significant difference (*p* < 0.05). A superscript value with the same letter indicates no significant difference between the samples. The value in parentheses indicates the standard deviation.

### 3.2. Microbial communities’ composition and diversity based on Illumina Miseq high-throughput sequencing

After sequence-quality filtering, a total of 2,768,608 rarefied reads were obtained from the V4 region of the bacterial 16S rRNA gene, 1,861,158 rarefied reads were obtained from the archaeal 16S rRNA gene, and 484,690 rarefied reads were obtained from fungal internal transcribed spacer (ITS) gene. Classification of OTUs at 97% similarity resulted in a total of 106,069, 41,418, and 15,398 OTUs for bacterial, archaeal, and fungal taxa, respectively. The number of OTUs at each classification level is shown in Supplementary Table S2.

A total of 41 bacterial phyla were identified in this study. The most common bacterial phyla were Actinobacteria (4.5–47.3%), Acidobacteria (6.9–30.2%), Chloroflexi (3.8–19.7%), Proteobacteria (4.0–47.2%), Gemmatimonadetes (9.7–23.2%), AD3 (0.01–18.7%), Planctomycetes (0.8–12.7%), and Firmicutes (0.1–33.1%; Supplementary Figure 1A). For archaea, only two phyla were detected in the terrace samples of this study (Supplementary Figure 1B). The communities in all stages were predominantly composed of a member of the phylum Crenarchaeota (41.0–97.7%), followed by Euryarchaeota (0.03–0.04%). Besides, there are many unannotated OTU sequences (2.2–58.9%). For fungi, a total of 13 phyla were identified, including Ascomycota (16.4–89.6%), Basidiomycota (1.3–20.9%), Mortierellomycota (0.02–17.5%), Rozellomycota (0.1–3.7%), and Entomophthoromycota (0–18.0%) (Supplementary Figure 1C).

#### 3.2.1. Differences in bacterial diversity among successional stages

The high-throughput sequencing results revealed that among bacterial communities across chronosequence, the dominant bacterial phyla were Proteobacteria, Acidobacteria, Chloroflexi, Actinobacteria, Planctomycetes, and Gemmatimonadetes (average content>5%). The abundance of Actinobacteria, Gemmatimonadetes, Nitrospirae, and Cyanobacteria was found to be significantly greater in the S0 stage of the terrace than in the other strata. The abundance of Nitrospirae gradually decreased as the terrace increased in altitude. In the S5 stage, the abundance of Proteobacteria and Planctomycetes is significantly less than that of other strata. However, they were most abundant in the middle S3 stage (Supplementary Figure 3).

The Linear Discriminant Analysis (LDA) Effect Size (LEfSe) was utilized to identify specialized communities in successional stages along the Ardley Island terrace. According to LEfSe multi-level species difference discriminant analysis, stage S0 contains more Hyphomicrobiales (Rhizobiales, heterotypic synonym) and Methyloligellaceae (Methylobacteriaceae) that are capable of methyl metabolism (Kelly et al., 2014; Oren and Xu, 2014; Jeong and Kim, 2019) than other strata, as well as Nitrosomonadaceae, Nitrospira, Nitrosospira, and Opitutus, which are related to nitrate and nitrite metabolism (Prosser et al., 2014; Supplementary Figure 4). Desulfarculales and Desulfosporosinus, which are sulfur metabolism-related bacteria, Myxococcales with predatory function, and Iamia, Haliangium, f__Ilumatobacteraceae.g__CL500-29_marine_group, o__Flavobacteriales.f__NS9_marine_group from marine sources.

Interestingly, in the S0 stratum, the bacteria that reproduce by budding and/or with numerous fibrillar appendages (Hirsch, 1974; Mujakić et al., 2022) were dominant, namely Gemmatimonadetes (for example, Gemmata, Pirellula), Planctomycetes (for example, Gemmataceae, Pirellulaceae, and Schlesneria), Alphaproteobacteria (Pedomicrobium and Bauldia). Acidicaldus, a sulfur-oxidizing, ferric iron-reducing acidophilic heterotrophic Proteobacterium, and Rhodoferax, with the capability of anoxygenic photoorganotrophy, anaerobic-dark fermentation, aerobic respiration, and anaerobic growth *via* sugar fermentation, are both enriched in the S1 stage. On the S3 and S1 stages, there are more Burkholderiaceae; the family includes true environmental saprophytic organisms, phytopathogens, opportunistic pathogens, as well as primary pathogens for humans and animals (Coenye, 2014). Stage S2 seems a lot like stage S4, and stage S1 is similar to stage S3. Most of microorganisms from the S5 stratum are parasitic or symbiotic with animals: Bacteroides, Prevotella, Alistipes, Agathobacter, Faecalibacterium, Subdoligranulum, Dialister, Megamonas, as well as cellulolytic bacteria Acidothermus.

BugBase was used to predict high-level bacterial phenotypic traits. It was found that the content of Gram-positive bacteria and motility factors in the S0 stage were much higher than those in other strata. S5 stage anaerobic and stress tolerance bacteria are much lower than other strata. Gram-negative bacteria and aerobic bacteria steadily increase as the successional stage rises. The ability to cope with stress and the potential to cause disease increases and subsequently decreases, indicating a hump condition (Supplementary Figure 5A).

The prediction of ecologically relevant functions of microbial taxa was carried out *via* a promising tool, FAPROTAX. As shown in Supplementary Figure 5B, methanotrophy, hydrocarbon_degradation, methylotrophy, aromatic_compound_degradation, nitrate_reduction, aerobic_nitrite_oxidation, nitrification, sulfate_respiration, predatory_or_exoparasitic, and chloroplasts in the S0 stratum are significantly higher than other strata. The S5 stage has significantly higher animal_parasites_or_symbionts, human pathogens, and cellulolysis than other strata. In each successional stage, photoheterotrophy and phototrophy increase and subsequently decrease. Nitrate_reduction, aerobic_nitrite_oxidation, and nitrification show a decreasing trend with the increase of stage, whereas animal_parasites_or_symbionts, nitrogen_fixation, cellulolysis, and chemoheterotrophy show an increasing trend as the stage increases. These function predictions are consistent with the unique bacterial characteristics of each stage in the LEfSe analysis.

#### 3.2.2. Differences in archaeal diversity among successional stages

High-throughput sequencing results showed that the terrace soil contained Thaumarchaeota, Euryarchaeota, and Crenarchaeota. Thaumarchaeota is the dominating archaea in all stages. Euryarchaeota has a higher abundance in the late and early stages. Crenarchaeota has a substantially larger abundance in the middle stage (Supplementary Figure 6). According to LEfSe multi-level species difference discriminant analysis, Candidatus_Nitrosocosmicus, Thermoplasmata and a large number of Marine_Group_II archaea were enriched in stage S0 (Supplementary Figure 7). Nitrososphaeraceae, unclassified Crenarchaeota, and unclassified Thaumarchaeota were enriched in the middle S1, S2, and S4 stages. In the highest S5 stage, Group_1_1c, and Euryarchaeota are more abundant.

#### 3.2.3. Differences In fungal diversity among successional stages

High-throughput sequencing results show that terrace soils mainly contain Ascomycota (47–81%) and Basidiomycota (5.9–7.7%). However, there was no significant difference in the content of other fungi in each stage, except for Glomeromycota, which was significantly less in the S1 stage than in other strata (Supplementary Figure 8). LEfSe analysis revealed that Rhizoctonia, unclassified genes of Verrucariaceae, and Hypocreaceae fungi were enriched in the bottom S0 stratum (Supplementary Figure 9). Among the middle stages, only the S3 stratum has characteristic fungal groups, including Nectriaceae, Yarrowia, unclassified genus of Didymellaceae, Hannaella, Glomeromycota, Entrophospora, Diversisporales, and Glomeromycetes. The highest S5 stage contains characteristic fungal groups: Capnodiales, Chaetothyriales and Herpotrichiellaceae.

### 3.3. Analysis of alpha diversity index

**Figure 3:**
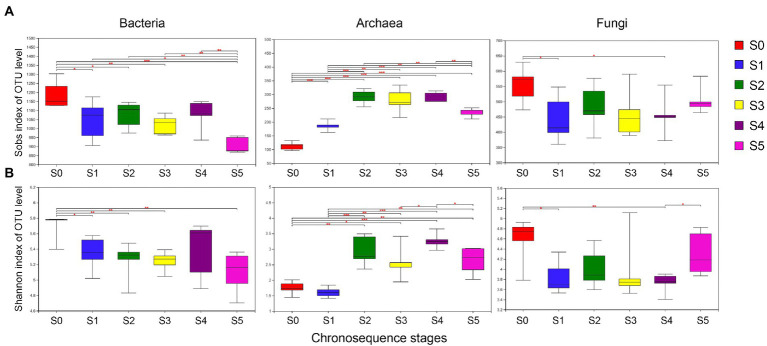
Alpha-diversity analyses of bacteria, archaea, and fungus along each successional stage. **(A)** Sobs diversity index, **(B)** Shannon diversity index.

In this study, the Sobs index and Shannon index were used to reflect the richness and diversity of microbial communities in different successional stages. As demonstrated in Figure 3, the late-stage S0 has much higher bacterial and fungal species richness (Sobs) and diversity (Shannon) than the other stages. However, the uppermost stratum S5 has significantly less bacterial species richness and diversity than the other strata. On the other hand, the richness and diversity of archaea in the S0 and S1 stages are lower than in other strata.

### 3.4. ANOSIM analysis

**Figure 4:**
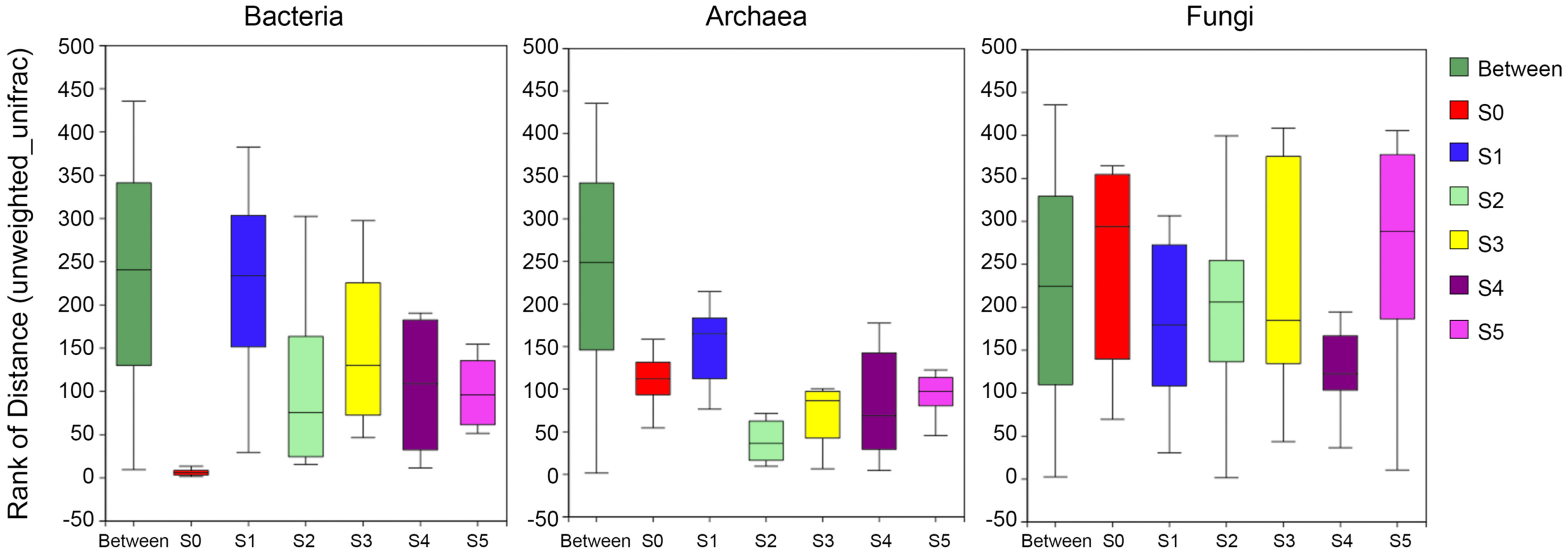
Analysis of similarities (ANOSIM) showing variation in bacterial, archaeal, and fungal community structure of different strata on the Antarctic Ardley Island terrace.

The ANOSIM/Adonis analysis (Figure 4) showed that the inter-group distances of the bacterial and archaeal communities were significantly greater than the intra-group distance (*R*-values were 0.60 and 0.67, respectively, and both *p*-values were 0.001); while the fungal communities were very heterogeneous among groups (*R*-value was 0.07, *p*-value was 0.13 > 0.05). The distribution of fungi in each stratum is quite uneven. In addition, the intra-group distances are greater than the inter-group distances.

### 3.5. Correlation analysis between environmental factors and microbial communities

**Figure 5:**
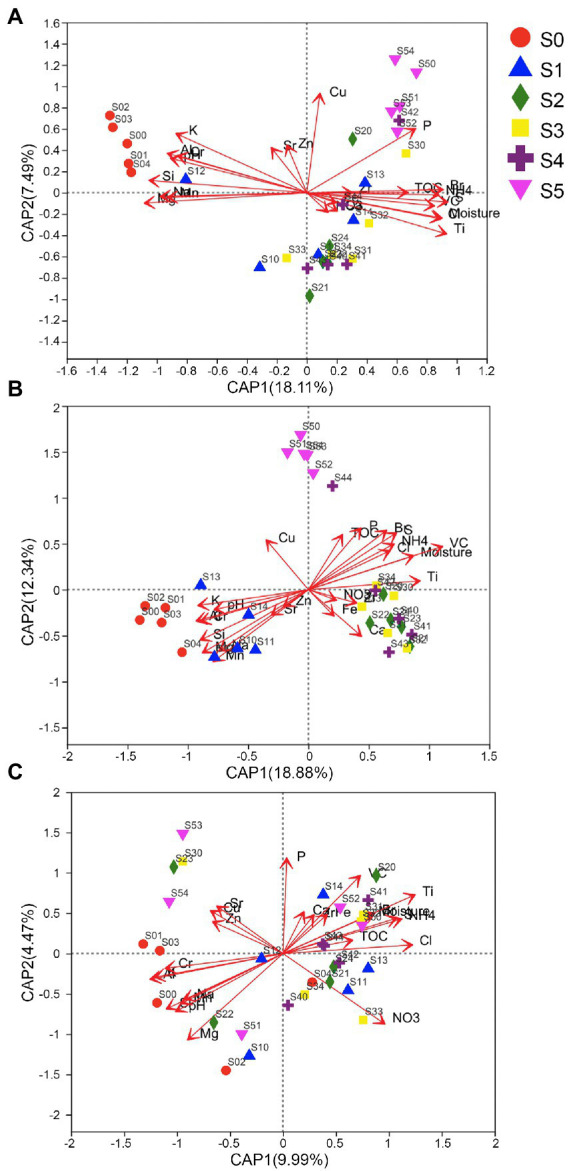
Distance-based redundancy analysis (db-RDA) analysis of **(A)** bacterial, **(B)** archaeal, and **(C)** fungal communities in various strata and environmental factors.

Db-RDA analyses were performed using these selected environmental factors. The communities of bacteria and archaea in the late stratum, as illustrated in Figure 5, are different from other strata. Communities in the middle stratum are mixed, but the early stratum (S5) can be distinguished from the other strata. On the other hand, the fungal communities in the S0 and S5 stages are indistinguishable, which might be due to substantial variances within the group. However, the fungal communities in the S0 and S5 stages can be distinguished from other middle stages and can be compared to other fungal communities using the Partial least squares-discriminant analysis (PLS-DA), which eliminates random variations within groups and reveals systematic differences between them (Supplementary Figure 2).

This was consistent with the results of PERMANOVA tests with Bray–Curtis distances that revealed that there were significant differences around the strata for prokaryotic 16S rRNA genes (Pseudo-*F* = 4.9 for the bacterial, and Pseudo-*F* = 5.2 for the archaeal community, both *p* = 0.001), but not for fungal ITS genes (Pseudo-*F* = 1.6, *p* = 0.097). PERMANOVA analysis showed that the bacterial and archaeal community structure in sample soils with different Mg, Si, and Na content was significantly different. The *R*^2^ values were 0.3334, 0.32783, and 0.28595, respectively for bacterial community, and 0.42117, 0.37815, and 0.36135, respectively for archaeal community. Therefore, the Mg, Si, and Na content were obviously related to bacterial and archaeal community structure changes. However, $NH_{4}^{+}$ content only was strong related to the archaeal community structure discrepancies (*R*^2^ values was 0.32655). On the other hand, Al, Ti, K, and Cl only were significantly related to the fungal community structure discrepancies (*R*^2^ values were 0.10736, 0.10672, 0.1041, and 0.09227, respectively) (Supplementary Table S3).

## 4. Discussion

The Ardley Island terrace is formed by the gradual uplift of the coast. It is speculated that the earliest nutrients came from marine sediments, followed by the nutrients brought by marine animals living on newly raised beaches, and then the humus produced by vegetation growth, and the nutrients brought by birds and human activities. Although previous studies have found that the plants in this terrace do not have obvious succession rules, our results show that the soil elements present obvious differences in various strata. The vegetation diversity of the Fildes Peninsula, Antarctica is not rich. The growth of the only vascular plant, *Deschampsia antarctica* (hair grass), was found not to have resulted from soil succession but from bird feces enrichment leading to a pseudo-succession in vegetation where fertilization gradients around bird colonies occur. There are no penguins settled on the terraces, and no *Deschampsia antarctica* is growing in this area. Since there are only lichens and mosses, higher plants were not observed in this area. Thus, there is no obvious plant succession. Besides, the elements, nutrients, organic matter, water-holding capacity and pH accumulated in each stratum are significantly different. Therefore, prokaryote communities in each successional stage are also significantly different.

### 4.1. Factors determining the microbial community distribution

Through PERMANOVA analysis, soil elements Mg, Si, and Na have a significant impact on the bacterial and archaeal community structure, while Al, Ti, K, and Cl only have a significant impact on the fungal community structure. These factors may be the key driving factors of the bacterial, archaeal and fungal communities assembly over the succession. On the other hand, environmental factors also have an important impact on the succession of microbial communities, which could be different among each microorganism. For instance, in addition to soil elements, the succession of bacterial communities is also greatly affected by pH and water content; archaeal communities are also greatly affected by $NH_{4}^{+}$; fungal communities are also affected by nutrients such as $NO_{3}^{-}$.

The S0 stage of the Ardley Island terrace is the latest stage to break away from the ocean during the geological uplift. Soil is still in the early stage of development. The bedrock and gravel are still in the weathering stage. Therefore, the soil element composition contains relatively high amounts of crust and rock composition. There are relatively abundant of P, S, and $NH_{4}^{+}$ in the S3, S4, and S5 stages, which may be because these stages are uplifted beaches from the ocean, and might be affected by marine animals, such as guano. It has experienced a long period of weathering and freeze–thaw process. In addition, the accumulation of nutrients and organic matter brought by marine animal activities and vegetation growth for a long time promotes the maturation of the upper soil on the terraces (Michel et al., 2014; Zhang et al., 2018). The soil in the upper stages (S3-S5) has higher water content and lower pH values than in the late stage (S0). The results are reasonable, since well-developed soil has a higher water storage capacity. In addition, the vegetation coverage, humic acid, and fulvic acid produced by mosses and lichens in the upper stages could decrease soil pH values (Rakusa-Suszczewski, 1993). High ammonia concentration produced during the decomposition of organic matter in the ocean could be another reason for higher pH value in the lower stratum.

Although many soil parameters are significantly correlated with fungal communities, in the db-RDA (Figure 5C) analysis, the fungal communities of each stage are not clearly clustered along the terrace. So, we speculated that other unknown distribution patterns or factors might influence fungal communities. For example, winds along the Antarctic coast affecting fungal spore dispersal; the uneven distribution patterns of fungal host plants resulted in the uneven distribution of fungal communities in soil; or some fungal species might be controlled by fungal biocontrol agents (mycoparasite). However, the bacterial and archaeal communities in the late stages are clearly distinguished from the other strata of the Ardley Island terrace.

The results of the correlation between microbial community structure and soil parameters from our study were in accordance with previous studies. In the study of Livingston island in the South Shetland Islands, it was found that soil total carbon, total nitrogen, and water content were the most significant factors affecting the distribution of bacteria (Ganzert et al., 2011). In Antarctica Valley, microbial communities were associated with soil K, C, and water content (Stomeo et al., 2012). Based on the previous studies and our results, the presence of available carbon, nitrogen, and water content in soil are considered as the driving factors that promote microbial growth along Antarctic chronosequence with extreme and oligotrophic conditions.

### 4.2. Characteristic microorganisms of each stratum and their succession

Through LEfSe analysis of the characteristic microorganisms on each stratum, it is found that the succession of microorganisms may be influenced by more complex and comprehensive factors, such as environmental instability, relationship with plants, ecological niches, and environmental tolerance.

#### 4.2.1. Bacterial community

Based on the soil element information, flora characteristics, and the correlation with environmental factors, it is found that the S0 stage has more marine characteristics, including the K, Na, and Mg elements related to its flora. In Antarctic surface soils, element concentrations are generally influenced by the element abundance of soil-forming basaltic and granitic rocks. Potassium was gradually released from the weathering potash feldspars, biotite, and muscovite micas. Part of sodium might derive from marine sources and moist air moves in from the polar oceans (Claridge and Campbell, 1977). High magnesium concentrations in the S0 stage probably derive from marine basaltic rocks (Malandrino et al., 2009). In addition, the study of soil development on marine terraces near Metaponto found that the trend of (Ca+Mg+K+Na)/Al ratios of the soils developed in the marine sediments supports the hypothesis of increasing terrace ages (Sauer et al., 2010). There was progressive feldspar weathering associated with element release and leaching in the late stage, which decreased with the increasing time uplifted to terrestrial.

Moreover, there are more marine-derived species in the flora. It is predicted that there are more motility factors, more Gram-positive bacteria in its flora, and more flora related to methane, nitrate, nitrite metabolism, and sulfur metabolism. It is speculated that this kind of flora can grow by using substances in marine sediments. Interestingly, there are a large number of filamentous appendages reproducing and budding strains in the S0 stratum. Unlike other stages, the S0 stage, the late stage uplifted to terrestrial, is still in an early stage of succession. Soils develop over time through a variety of interrelated processes, such as, organic enrichment, leaching of soluble salts, translocation of clay minerals, and changes in pH (Schmid, 2013). Thus, the ecology in this stage is considered as unstable and barren environments. Appendage and budding bacteria are easy to accumulate, which is conducive to resisting damage and obtaining nutrients in a rapidly changing and barren environment. Actinobacteria have a competitive advantage over other microorganisms due to their resilience and adaptability which help them to survive under harsh circumstances (Shivlata and Satyanarayana, 2015). Budding bacteria, Gemmatimonadetes, live in various harsh environments and are frequently resistant to the stress conditions occurring in their habitat. They are well-adapted to low-moisture environments and strong tolerance to the barren environment (DeBruyn et al., 2011). In addition, they could be found in extremely oligotrophic environments as well, for instance, on the surface of cave walls (Zhou et al., 2007; Pašić et al., 2010) and weathering rocks (Cockell et al., 2009). Aggregation or attachment is particularly beneficial in habitats with low nutrient concentrations or a fluctuating nutrient source (Hirsch, 1974).

The highest level, the S5 stratum with the longest succession time, is related to phosphorus, which may be related to animal feces. Moreover, there are a larger number of animal parasites_or_symbionts, human pathogens, and cellulolytic strains may be due to the fact that this stage has been uplifted from the sea for the longest time. The flora is more affected by terrestrial animals, humans and plants. During the succession of the S0–S5 stage of bacteria, the bacteria that use photosynthesis to obtain organic carbon gradually increase and reach the maximum amount in the middle stage. However, with the growth of vegetation and the accumulation of organic matter in the soil, the photosynthetic bacteria decrease, but the chemoautotrophy function is always the main metabolic mode of the flora and it gradually increases with succession.

#### 4.2.2. Archaeal community

When analyzing the archaea’s characteristics in each stage, we discovered very interesting phenomena. Enriched Candidatus Nitrosocosmicus in the late stage, enriched Nitrososphaeraceae in the middle stage, and enriched Group 1.1c in the early stage, are all the members of the Thaumarchaeota, but with different capabilities of Ammonia-oxidizing. Thaumarchaeota is a widely dispersed archaeal phylum that includes both ammonia-oxidizing archaea (AOA) and other archaeal species that have not been shown to oxidize ammonia (including Group 1.1c and Group 1.3).

In stage S0, Candidatus_Nitrosocosmicus, Thermoplasmata, and a large number of Marine_Group_II archaea were enriched. Candidatus Nitrosocosmicus, the ammonia-oxidizing archaeon is distinguished by its tolerance to high ammonia concentrations (Lehtovirta-Morley et al., 2016; Liu et al., 2021). Members of the Thermoplasmata class have been identified as methylotrophic methanogens and significant drivers in the carbon cycle in both marine and freshwater sediments (Compte-Port et al., 2020). Marine Group II, the most widespread marine planktonic archaea with photoheterotrophic lifestyles based on proteorhodopsin, has been discovered in all the world’s oceans, from surface waters to the deep sea. It can be observed that the archaea in the S0 stage are more affected by marine microorganisms.

Prior to the identification of “Candidatus Nitrosocosmicus,” the ammonia-oxidizing members of Thaumarchaeota were thought to be ammonia-sensitive (Oton et al., 2016; Liu et al., 2021). Candidatus Nitrosocosmicus, on the other hand, is characterized by its tolerance to high ammonia concentrations (Lehtovirta-Morley et al., 2016; Liu et al., 2021). However, Ammonia-oxidizing archaea (AOA), Nitrososphaeraceae are considered adaptation to low ammonia concentrations, and an autotrophic or possibly mixotrophic lifestyle, and may play an important role in nitrogen removal (Stieglmeier et al., 2014). Group 1.1c Thaumarchaeota are the most common archaeal group found in acidic forest soils. They are extensively distributed, especially in conditions with higher moisture and organic matter content (Oton et al., 2016). According to studies of the foreland of the receding Rotmoosferner glacier in the Austrian Central Alps, Crenarchaeal communities in the soil in different stages of development are distinct from each other. In contrast, Group 1.1c Thaumarchaeota are only found in mature soils (Nicol et al., 2006).

The previous study revealed that marine sediments have high ammonia concentrations produced during the decomposition of organic matter in the ocean (Mackin and Aller, 1984). It is speculated that high-ammonia tolerant archaea might originate from the ocean, and still retains as dominant ammonia-oxidizing archaea (AOA) in the S0 stage, then decreases gradually with the succession. Then, the AOA with the ability to utilize low-concentration ammonia became dominant in the soil with a low ammonia concentration. Final, Group 1.1c, which could eventually utilize organic matter rather than ammonia (Weber et al., 2015), dominated later in succession.

#### 4.2.3. Fungal community

Unlike prokaryotic microorganisms, the distribution of fungi in each stratum is quite uneven, resulting in significant communication variances in each stratum. The intra-group distances are greater than the inter-group distances. It is speculated that this is related to the uneven distribution of fungal symbiosis or parasitic plants. The late, middle, and early stages of terrace succession still have their own characteristic fungi. Moreover, several plant symbiotic fungi were found in successional stages. It is speculated that the uneven distribution of fungal host plants (encrusted lichens and mosses) might result in the uneven distribution of fungal communities in the soil during sampling. Some fungal species might be controlled by fungal biocontrol agents (mycoparasite). Besides, the sea breeze is possible factor that might influence fungal spore dispersal.

Plant pathogens, Fungicolous fungi, and lichenized fungi were enriched in the late stage. It is speculated that there are some unique lichen species in this stage. Although there are reports of this stage of lichen in the prior study, some can only be defined as genera, but not specific species. Rhizoctonia organisms are often soiled fungi mostly associated with roots and usually pathogens. However, there have been observations of saprophytic and symbiotic species. A few species have been identified as parasitic on herbaceous plants or bryophytes (mosses) (Hietala et al., 2001). The Verrucariaceae (Ascomycota) is a lichenized fungus family with a broad group of algal symbionts, including certain algae that are seldom or never associated with other lichens (Thüs et al., 2011). Hypocreaceae members are fungicolous on fungi solely or largely, and have been widely investigated and commercialized as biocontrol agents (Põldmaa, 2000). Fungicolous fungi are a huge, varied, ecologically, and a trophic group of fungal-associated organisms. Symbionts, mycoparasites, saprotrophs, and even neutrals are all terms used to describe them (Sun et al., 2019).

The ascomycete family Nectriaceae (Hypocreales) were enriched in the middle stage (S3 stage). Hypocreales is one of the most successful orders of ascomycetes on mosses and hepatics. More than 30 species of Bionectriaceae and Nectriaceae are obligately bryophilous (Döbbeler, 2005). The yeast Yarrowia is extensively dispersed and found in Antarctic marine sediments (Zhang et al., 2012). Hannaella is a genus of basidiomycetous yeast that belongs to the Tremellales order of the phylum Basidiomycota. This genus currently has around 12 species, all of which are prevalently spread on the leaf surfaces of numerous plants, such as rice, wheat, and fruit trees (Li et al., 2021). Although no species of this genus are currently found in polar regions, a former member of this genus, *Cryptococcus luteolus* (*Hannaella luteola*), has been discovered in Antarctic soils samples from the Capes Evans-Royds area, and the Ross Dependency (di Menna, 1966; Atlas et al., 1978). It was also found in soil samples from non-polar cold habitats of Asia; the Pamir Mountains (Babjeva and Reshetova, 1971). Despite the lack of evidence, it does not rule out that this yeast is related to moss. The ancestral species in Didymellaceae are the Graminae-pathogens, *Ascochyta hordei*, and *Phoma paspali*. In Australia and New Zealand, the latter species has long been thought to be an indigenous grass pathogen. A large number of Phoma genera of Didymellaceae have also been found in the mosses of Antarctica (McRae and Seppelt, 1999; Aveskamp et al., 2010). In addition, Entrophospora, Diversisporales, and Glomeromycota enriched in the S3 stage were known as arbuscular mycorrhizal fungi (AMF), one of the most widely distributed plant symbiotic fungi in nature (Wu et al., 2013; Camenzind et al., 2014; Qi et al., 2020). Glomeromycota can form arbuscular mycorrhizas with a huge number of plants: liverworts, ferns, gymnosperms, and angiosperms (Bonfante and Venice, 2020). It might form arbuscular mycorrhizas with the thalli of bryophytes in biological soil crusts (BSC) to profit from each other. The host plant’s photosynthetic carbohydrates are redirected to AM’s growth. Water and nutrient uptake from the fungal partner to the host plant are exchanged.

In most cases, AM symbiosis benefits the host plant by improving plant growth, nitrogen absorption, drought tolerance, and soil structure. Besides, it may have helped plants adapt from marine to terrestrial environments. This is crucial for the restoration and succession of the vegetation on the raised coastline.

In the early stage, melanized fungi were enriched. The members of Herpotrichiellaceae are also known as animal pathogens. In certain harsh conditions, such as Antarctic rocks, melanized microbes are typically the dominant species, indicating that melanin is helpful to their life cycle (Horré and De Hoog, 1999; Chowdhary et al., 2015; Abdollahzadeh et al., 2020). One of the essential parts of their extraordinary ability to resist external stressors is the structure of their cell wall. The cells are encased in a thick, strongly melanized cell wall coated with black hard plaques, providing supplementary protection and making them essentially impenetrable and resistant to commercial enzymes such as chitinases and glucanases (Selbmann et al., 2012). The ability of the melanized fungus to withstand cosmic and terrestrial ionizing radiation shows that melanin is also important for radioprotection. Furthermore, melanized fungal species, such as those found in Chernobyl’s reactor, respond to ionizing radiation with enhanced growth (Dadachova and Casadevall, 2008). The melanized fungi include saprobes, plant and human pathogens, mycoparasites, rock-inhabiting fungi (RIF), lichenised, epi-, ecto-, and endophytes. It is speculated that these characteristic groups may be tolerant to harsh environments and can form biofilms (subaerial biofilms forming microorganisms; SAB), inhabit rocks, and weather rocks. Other microorganisms or plants may be able to colonize and thrive as a result of these functions. For instance, the formation of biofilms is conducive to the colonization and production of other microorganisms, and the weathering rock is conducive to the growth of lichens and other plants (Abdollahzadeh et al., 2020).

The enriched fungi along the Ardley Island terrace in accordance with the results of soil development and plant succession in this area. The studies of Jens Boy and Robert Godoy concluded that: “All temporal gradients showed soil development leading to differentiation of soil horizons, carbon accumulation and increasing pH with age. Photoautotroph succession occurred rapidly after glacier retreat, but occurrences of mosses and lichens interacting with soils by rhizoids or rhizines were only observed in the later stages. The community of ground-dwelling mosses and lichens is the climax community of soil succession, as the Antarctic hairgrass *Deschampsia antarctica* was restricted to ornithic soils. Neither *D*. *antarctica* nor mosses at the best-developed soils showed any sign of mycorrhization.” (Boy et al., 2016). As excess of nutrients is known to inhibit mycorrhization (de Vries et al., 2007).

Perhaps the functional succession of fungi is: degradation and utilization of plants as nutrients (saprophytic fungi) – benefit from symbiosis with plants (AMF) – rock-inhabiting to help vegetation colonize rocks, resist stress (melanized fungi), and infection animals as pathogens.

## 5. Conclusion

The Ardley Island terrace is a typical coastal uplift terrace with a complete and well-aged six strata. Because of the slow growth of organisms in Antarctica, our results showed that although this area spans over 200–7,200 years, the succession of the microbial community can still be detected. The different historical and geological background of each successional stage is the fundamental reason for the different microbial community composition. The different strata heights correspond to the time gradient of the coastal uplift. PERMANOVA tests revealed that soil elements Mg, Si, and Na were related to the bacterial and archaeal community structure discrepancies, and Al, Ti, K, and Cl were related to the fungal community structure discrepancies. These soil elements may be the main driving factors for the succession of bacteria, archaea and fungi along terrace. However, environmental factors also have an important impact on the succession of microbial communities, which may differently depend on the microorganism. Some marine microbial groups were found in near-coastal soils of the S0 stratum. At the highest level with the longest succession period, animal pathogenic bacteria and microorganisms that may be more resistant to stress appeared. The photosynthetic bacteria, archaea that can perform ammonia oxidation at low concentrations of ammonium salts, and arbuscular mycorrhizal fungi (AMF), which are most related to soil nutrition, are in the middle strata. This also corresponds to the middle stratum is most related to ammonium salt, nitrate, and TOC in PCoA results. Moreover, the analysis of the characteristic microorganisms along the terrace reveals that the succession of microorganisms may be influenced by more complex and comprehensive factors, such as environmental instability, relationship with plants and ecological niches, and environmental tolerance. Interestingly, we found that a large number of budding reproduction and/or with filamentous appendages bacteria were enriched in the S0 stratum, which might be related to its adaptation to rapid changes and barren environments. Another is the succession of Thaumarchaeota archaea in different classes that began from: ammonia-oxidizing archaea (AOA) with the ability to utilize high-concentration ammonia to AOA with the ability to utilize low-concentration ammonia, and lastly to AOA with the inability to utilize ammonia. The other is the changes in the fungi-plants relationship in different classes (saprophytic-symbiosis-mutualism). In short, the clear chronological sequence of Ardley Island terrace (Antarctica), less disturbed by animals, plants, and humans, and the obvious microbial succession, made it an ideal place to study the succession of microbial communities.

## Data availability statement

The datasets presented in this study can be found in online repositories. The names of the repository/repositories and accession number(s) can be found below: NCBI – PRJNA784744.

## Author contributions

PN, YZ, and FP designed the experiment and supervised all work. LY was involved in the experiment of principal component analysis. PN and YZ were involved in the field work and the sequencing data analysis. All authors contributed to the article and approved the submitted version.

## Funding

This work was supported by the National Key R&D Program of China (2022YFC2807500) the R and D Infrastructure and Facility Development Programme of the Ministry of Science and Technology of the People’s Republic of China (Grant number NIMR-2020-8), the National Natural Science Foundation of China (Grant number 42076230), and the Chinese Polar Scientific Strategy Research Fund (IC201706).

## Conflict of interest

The authors declare that the research was conducted in the absence of any commercial or financial relationships that could be construed as a potential conflict of interest.

## Publisher’s note

All claims expressed in this article are solely those of the authors and do not necessarily represent those of their affiliated organizations, or those of the publisher, the editors and the reviewers. Any product that may be evaluated in this article, or claim that may be made by its manufacturer, is not guaranteed or endorsed by the publisher.

## Footnotes

^1^ http://www.drive5.com/usearch/

^2^ https://bugbase.cs.umn.edu/index.html
