# Supplementary material for: Antarctic Ardley Island terrace — An ideal place to study the marine to terrestrial succession of microbial communities
Source: Front Microbiol. 2023 Feb 6;14:942428. doi: 10.3389/fmicb.2023.942428 (PMC9940900; doi:10.3389/fmicb.2023.942428)
Supplement: Supplementary file 1 [file Data_Sheet_1.ZIP › 01 SuppInfo S1_Data Sheet.docx]

Supplementary Material

Methods

1. **Sampling sites**

Supplementary Material should be uploaded separately on submission. Please include any supplementary data, figures and/or tables. All supplementary files are deposited to FigShare for permanent storage and receive a DOI.

| **Sampling site** | **GPS** | **Height (m)** | **Soil temperature** | **pH** | **Water content** |
| --- | --- | --- | --- | --- | --- |
| S0-0 | 62º12'44.7''S 58º56'50.0''W | 6.9 | 7.0 | 6.73 | 15.0% |
| S0-1 | 62º12'39.3''S 58º56'51.2''W | 9.6 | 7.0 | 6.80 | 11.6% |
| S0-2 | 62º12'42.0''S 58º56'51.5''W | 6.2 | 7.0 | 6.80 | 15.9% |
| S0-3 | 62º12'46.0''S 58º56'48.9''W | 10 | 7.0 | 6.69 | 22.2% |
| S0-4 | 62º12'47.5''S 58º56'44.9''W | 14 | 6.5 | 6.70 | 9.2% |
| S1-0 | 62º12'45.9''S 58º56'46.2''W | 12 | 4.2 | 6.11 | 29.3% |
| S1-1 | 62º12'43.9''S 58º56'48.9''W | 13 | 6.0 | 5.36 | 42.3% |
| S1-2 | 62º12'44.5''S 58º56'47.1''W | 13 | 6.1 | 6.50 | 29.3% |
| S1-3 | 62º12'46.4''S 58º56'45.1''W | 15 | 5.5 | 5.56 | 32.2% |
| S1-4 | 62º12'46.7''S 58º56'42.0''W | 13 | 6.5 | 5.82 | 27.4% |
| S2-0 | 62º12'45.7''S 58º56'42.6''W | 15 | 3.1 | 6.06 | 32.3% |
| S2-1 | 62º12'45.0''S 58º56'44.3''W | 16 | 4.5 | 5.75 | 44.2% |
| S2-2 | 62º12'45.4''S 58º56'43.4''W | 14 | 3.8 | 5.96 | 42.9% |
| S2-3 | 62º12'45.9''S 58º56'40.3''W | 11 | 3.5 | 5.95 | 30.0% |
| S2-4 | 62º12'46.8''S 58º56'37.9''W | 19 | 4.9 | 5.96 | 40.2% |
| S3-0 | 62º12'44.6''S 58º56'41.0''W | 19 | 3.5 | 5.05 | 36.1% |
| S3-1 | 62º12'44.0''S 58º56'43.1''W | 21 | 4.8 | 6.05 | 27.9% |
| S3-2 | 62º12'44.4''S 58º56'42.0''W | 11 | 4.0 | 5.45 | 43.7% |
| S3-3 | 62º12'45.1''S 58º56'39.2''W | 22 | 3.8 | 5.45 | 27.7% |
| S3-4 | 62º12'46.2''S 58º56'37.3''W | 12 | 3.5 | 6.15 | 40.3% |
| S4-0 | 62º12'43.8''S 58º56'40.4''W | 13 | 5.1 | 5.80 | 46.1% |
| S4-1 | 62º12'44.0''S 58º56'33.0''W | 23 | 4.8 | 5.75 | 51.9% |
| S4-2 | 62º12'43.4''S 58º56'42.1''W | 24 | 4.8 | 6.28 | 32.1% |
| S4-3 | 62º12'44.7''S 58º56'38.0''W | 21 | 4.5 | 5.75 | 32.9% |
| S4-4 | 62º12'45.6''S 58º56'36.7''W | 16 | 4.0 | 5.85 | 33.3% |
| S5-0 | 62º12'40.7''S 58º56'45.1''W | 20 | 5.0 | 6.13 | 38.1% |
| S5-1 | 62º12'44.3''S 58º56'38.8''W | 21 | 7.3 | 5.54 | 48.2% |
| S5-2 | 62º12'40.0''S 58º56'46.0''W | 23 | 4.5 | 5.73 | 36.9% |
| S5-3 | 62º12'42.0''S 58º56'40.4''W | 23 | 7.5 | 5.93 | 42.4% |
| S5-4 | 62º12'42.9''S 58º56'35.9''W | 21 | 4.5 | 5.72 | 42.7% |

# Illumina high-throughput sequencing

## Bacterial 16S rRNA gene analysis

The bacterial hypervariable V4 region of the 16S rRNA genes was amplified using the universal primers 515F (5’-GTGCCAGCMGCCGCGGTAA-3’) with a 7-nucleotide barcode and 907R (5’-CCGTCAATTCMTTTRAGTTT-3’) (Stubner, 2002).The thermal cycler protocol consisted of an initial denaturation at 98℃ for 30 s, followed by 25 cycles of denaturation at 98℃ for 15 s, annealing at 50℃ for 30 s, extension at 72℃ for 30 s, and a final extension at 72℃ for 5 min.

## Archaeal 16S rRNA gene analysis

The archaeal V5-6 regions of 16S rRNA genes were amplified using primers 524F-10-extF (5’-TGYCAGCCGCCGCGGTAA-3’) with a 7-nucleotide barcode and Arch958-modR (5’-YCCGGCGTTGAVTCCAATT-3’) (Pires et al., 2012). The thermal cycler protocol consisted of an initial denaturation at 95℃ for 30 s, followed by 25 cycles of denaturation at 95℃ for 15 s, annealing at 55℃ for 30 s, extension at 72℃ for 30 s, and a final extension at 72℃ for 5 min.

## Fungal ITS gene analysis

The fungal rDNA ITS1-5.8S-ITS2 region was amplified using primers ITS5 (5’-GGAAGTAAAAGTCGTAACAAGG-3’) with a 7-nucleotide barcode and ITS4 (5’-TCCTCCGCTTATTGATATGC-3’) (Baldwin, 1992). Amplifications of the fungal ITS regions were performed, consisting of an initial denaturation at 95℃ for 30 s, followed by 25 cycles of denaturation at 95℃ for 15 s, annealing at 50℃ for 30 s, extension at 72℃ for 30 s, and a final extension at 72℃ for 5 min.

The PCR reactions were performed in 25 μL reaction mixture containing 0.25 μl Q5 high-fidelity DNA polymerase (NEB), 5 μL reaction buffer, 5 μL high GC buffer, 0.5 μL of 10 mM dNTP, 1 μL template DNA, 1 μL of each primer (10 μM), and 11.25 μL sterile double-distilled H_2_O.

PCR products were purified using an AxyPreDNA Gel Extraction Kit (Axygen Biosciences, Corning, NY, USA) following to the manufacturer’s instructions. The purified PCR amplicons from each sample were then mixed after quantification using a Quant-iT PicoGreen dsDNA Assay Kit (Invitrogen) in the Microplate reader (Bio Tek, FLx800). Sequencing was performed on the Illumina Miseq Platform.

High-quality clean reads were achieved by removing short reads (<150 bp), long homopolymers (> 8 bp), and reads with ambiguous bases using QIIME software v. 1.8.0 (Boulder, CO, USA). Chimera sequence checking was performed and eliminated by USEARCH v. 5.2.236 (http://www.drive5.com/usearch/). These reads were clustered into OTUs based on 97% sequence similarity using UCLUST, and the most abundant sequence was selected as representative of each OTU. The OTUs with sequence numbers lower than 0.001% of total sequences were removed (Bokulich et al., 2013). The resulting OTU sequences were then aligned with the Greengenes database release 13.8 for bacterial and archaeal 16S rRNA sequences (DeSantis et al., 2006) and the UNITE database release 5.0 for fungal ITS region sequences (Kõljalg et al., 2013).

1. **References**

Baldwin, B. G. (1992). Phylogenetic utility of the internal transcribed spacers of nuclear ribosomal DNA in plants: An example from the compositae. *Mol. Phylogenet. Evol.* 1, 3–16. doi:10.1016/1055-7903(92)90030-K.

Bokulich, N. A., Subramanian, S., Faith, J. J., Gevers, D., Gordon, J. I., Knight, R., et al. (2013). Quality-filtering vastly improves diversity estimates from Illumina amplicon sequencing. *Nat. Methods* 10, 57–59. doi:10.1038/nmeth.2276.

DeSantis, T. Z., Hugenholtz, P., Larsen, N., Rojas, M., Brodie, E. L., Keller, K., et al. (2006). Greengenes, a chimera-checked 16S rRNA gene database and workbench compatible with ARB. *Appl. Environ. Microbiol.* 72, 5069–5072. doi:10.1128/AEM.03006-05.

Kõljalg, U., Nilsson, R. H., Abarenkov, K., Tedersoo, L., Taylor, A. F. S., Bahram, M., et al. (2013). Towards a unified paradigm for sequence-based identification of fungi. *Mol. Ecol.* 22, 5271–5277. doi:10.1111/mec.12481.

Pires, A. C. C., Cleary, D. F. R., Almeida, A., Cunha, Â., Dealtry, S., Mendonça-Hagler, L. C. S., et al. (2012). Denaturing gradient gel electrophoresis and barcoded pyrosequencing reveal unprecedented archaeal diversity in mangrove sediment and rhizosphere samples. *Appl. Environ. Microbiol.* 78, 5520–5528. doi:10.1128/AEM.00386-12.

Stubner, S. (2002). Enumeration of 16S rDNA of Desulfotomaculum lineage 1 in rice field soil by real-time PCR with SybrGreen^TM^ detection. *J. Microbiol. Methods* 50, 155–164. doi:10.1016/S0167-7012(02)00024-6.
