# Supplementary material for: Antarctic Ardley Island terrace — An ideal place to study the marine to terrestrial succession of microbial communities
Source: Front Microbiol. 2023 Feb 6;14:942428. doi: 10.3389/fmicb.2023.942428 (PMC9940900; doi:10.3389/fmicb.2023.942428)
Supplement: Supplementary file 2 [file Data_Sheet_2.PDF]

Supplementary Material

Figure

(A)

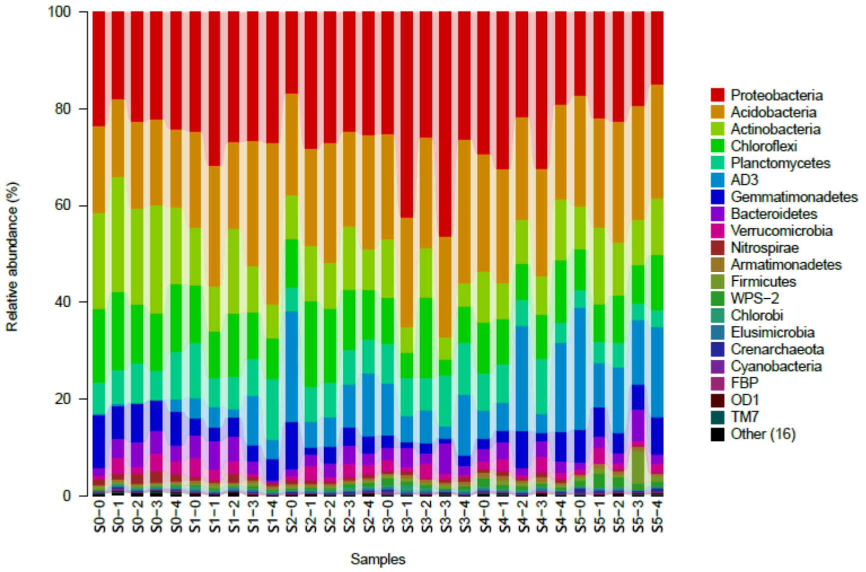

(B)

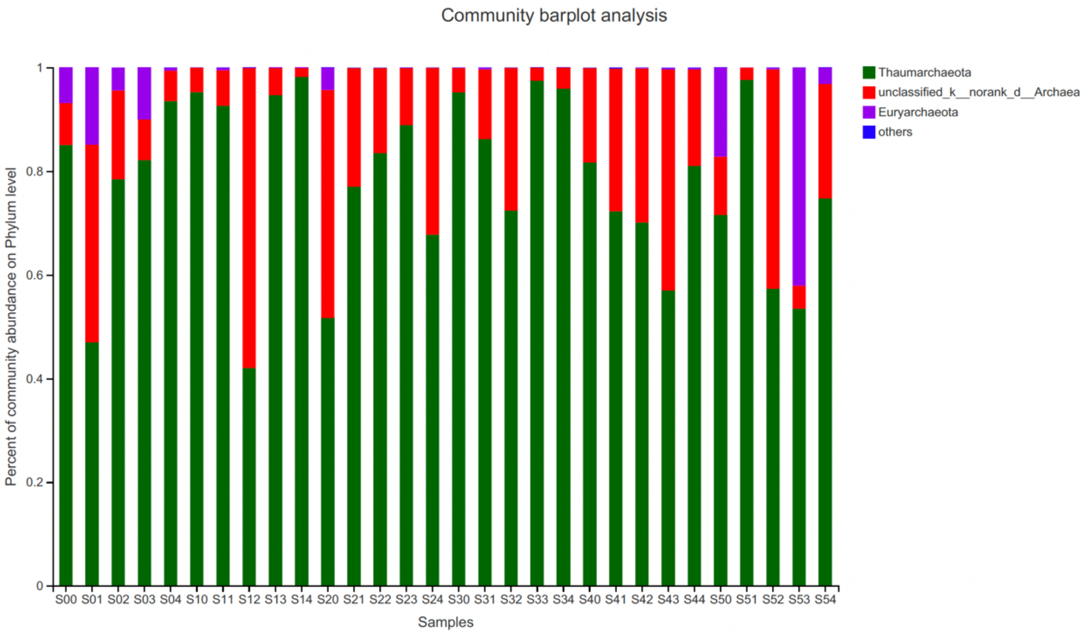

(C)

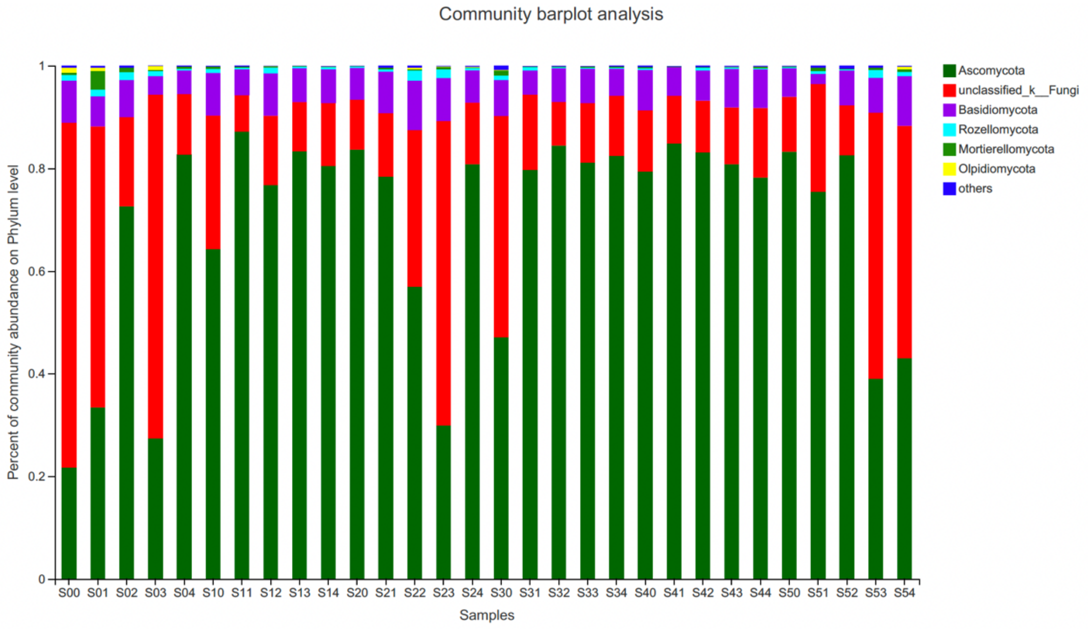

Supplementary Figure 1. Microbial communities composition in terrace soils shown as relative abundance (%) of taxa at the phylum level. **(A)** Bacterial community composition, **(B)** Archaeal community composition, **(C)** Fungal community composition.

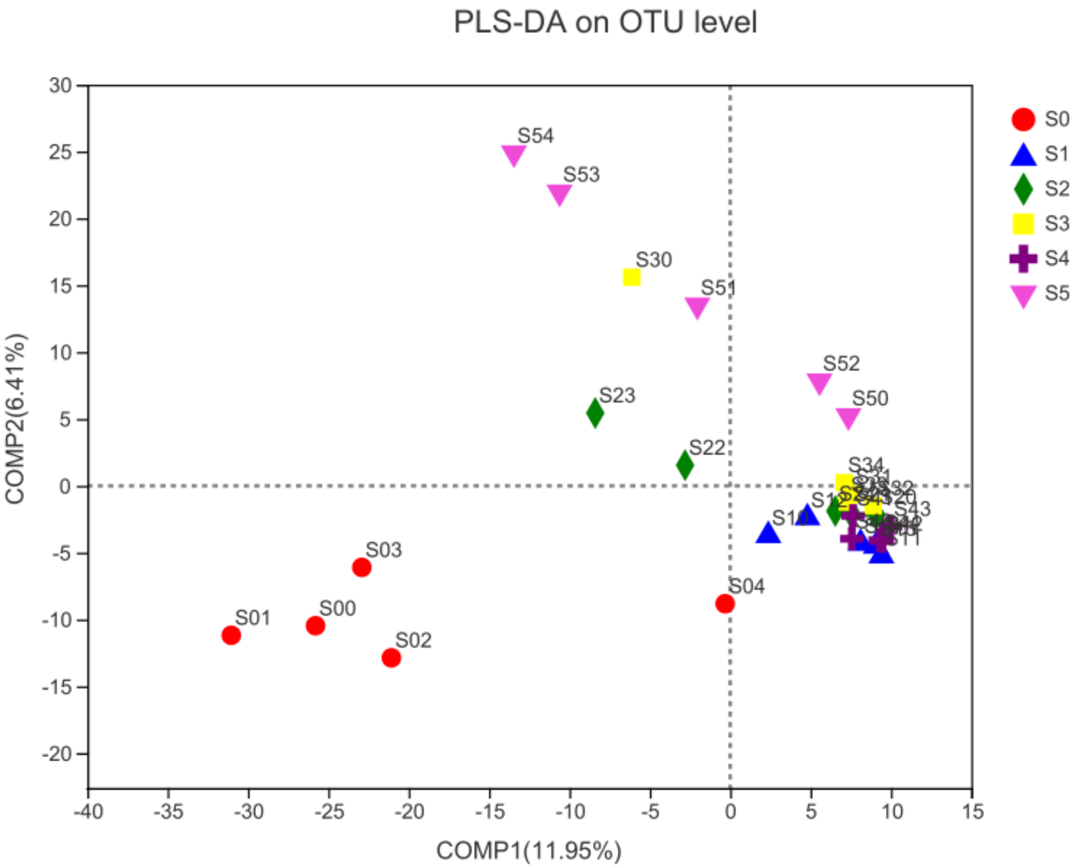

Supplementary Figure 2. Partial least squares-discriminant analysis (PLS-DA) of fungal communities

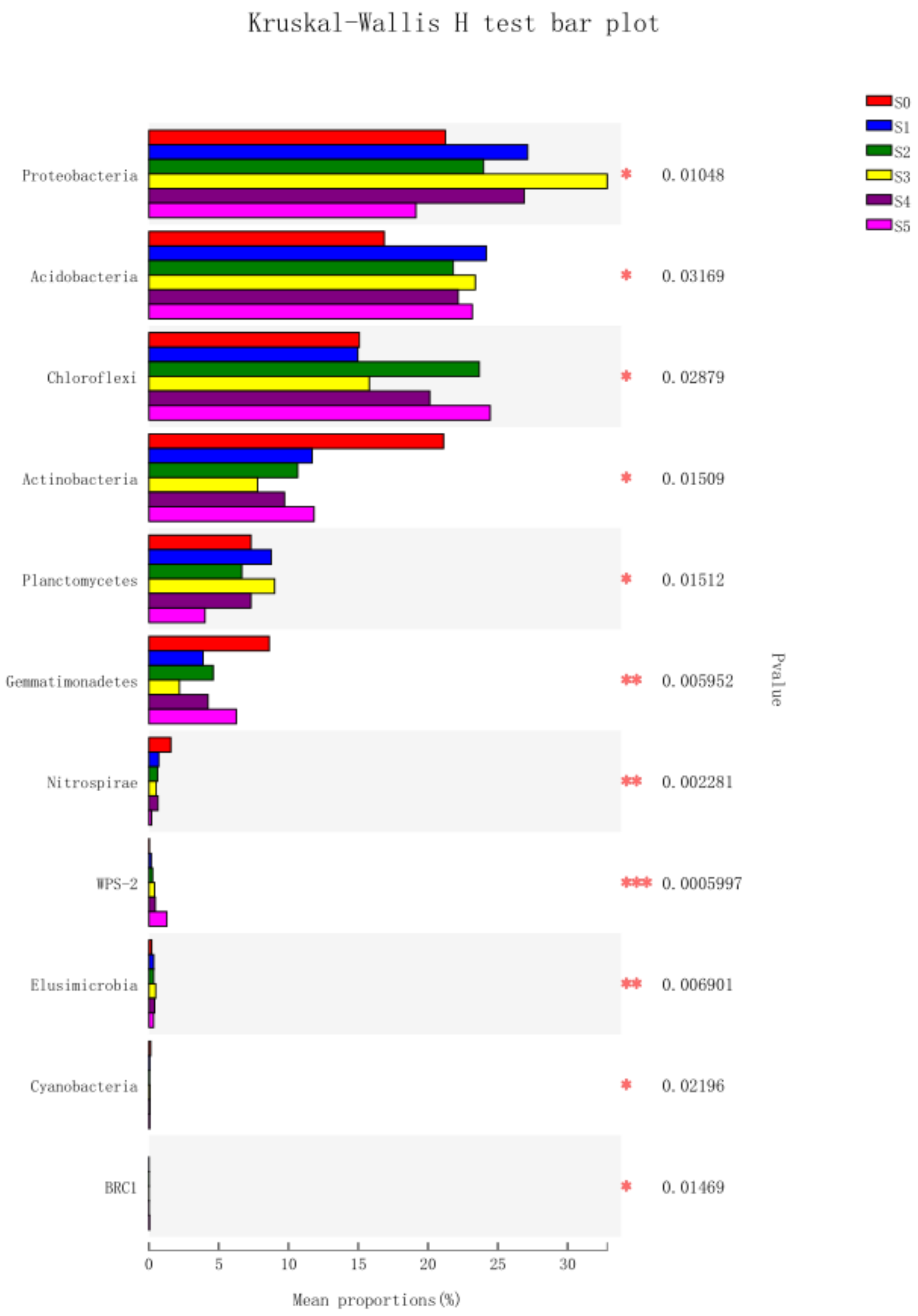

Supplementary Figure 3. Differential bacterial phyla levels comparison between different successional stages. * 0.01 < P ≤ 0.05, ** 0.001 < P ≤ 0.01, and *** P ≤ 0.001.

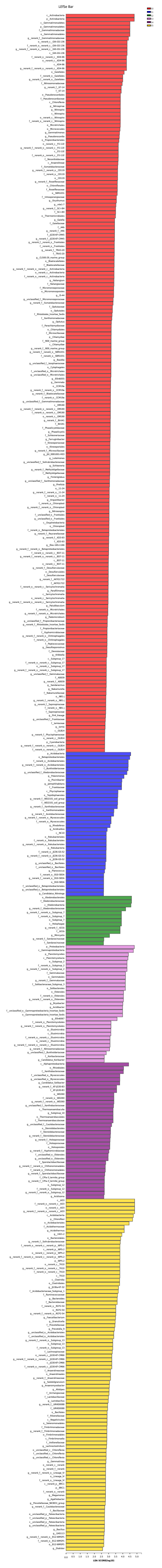

Supplementary Figure 4. LEfSe analysis. The histogram of the Linear discriminant analysis (LDA) scores illustrates the differentially abundant bacterial communities along successional stages.

(A) **(B)**

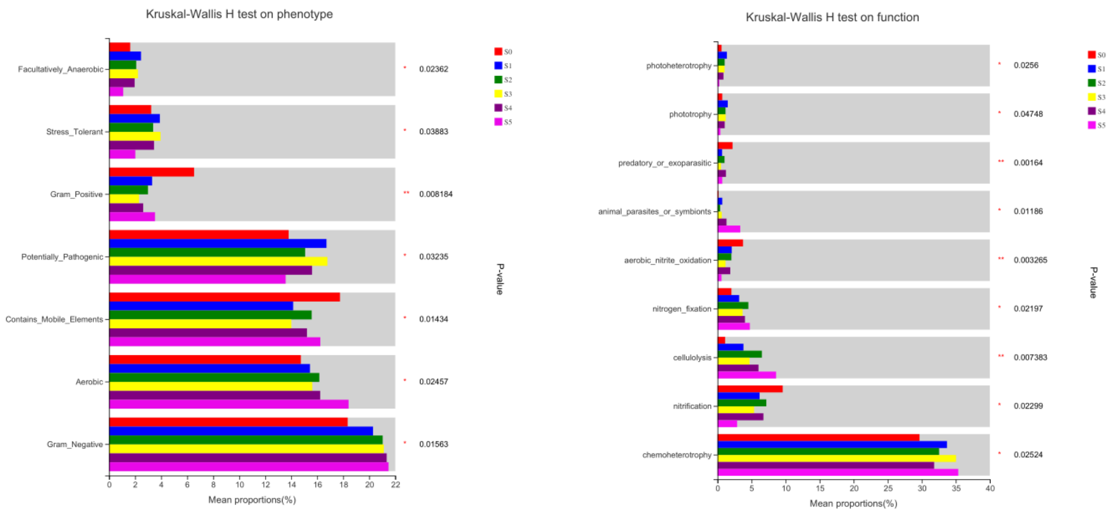

Supplementary Figure 5. Bacterial community phenotype and function prediction. **(A)** BugBase bacterial community phenotype prediction and difference detection, **(B)** Prediction of bacterial community functions based on FAPROTAX database. * 0.01 < P ≤ 0.05, ** 0.001 < P ≤ 0.01, and *** P ≤ 0.001.

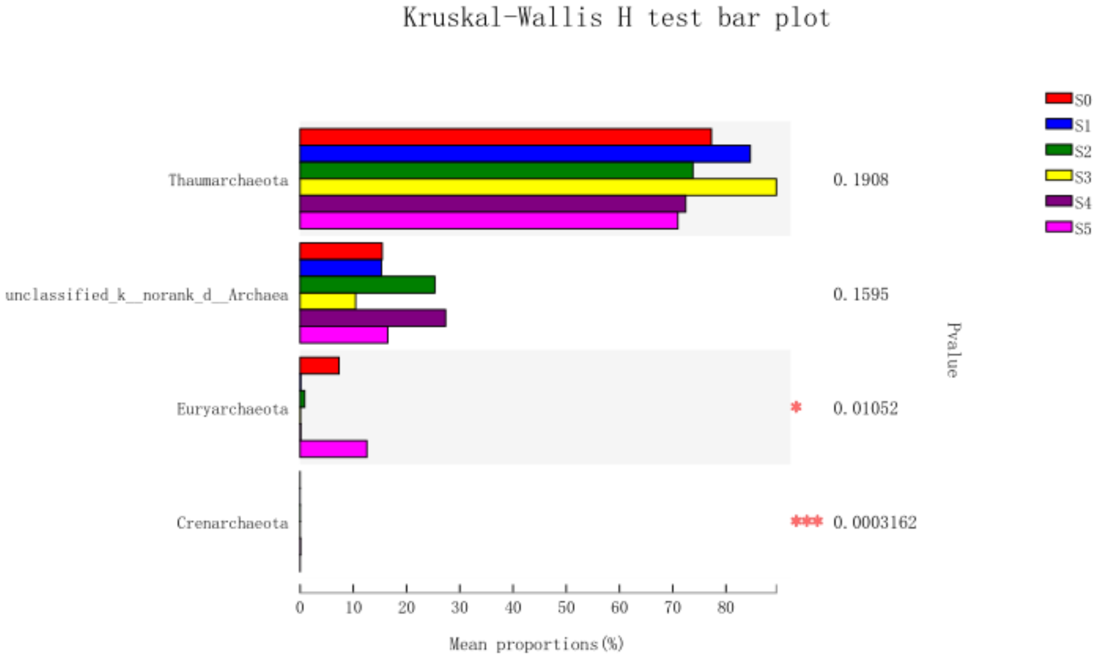

Supplementary Figure 6. Differential archaeal phyla levels comparison between different successional stages. * 0.01 < $P \leq 0.05$, ** 0.001 < $P \leq 0.01$, and *** $P \leq 0.001$.

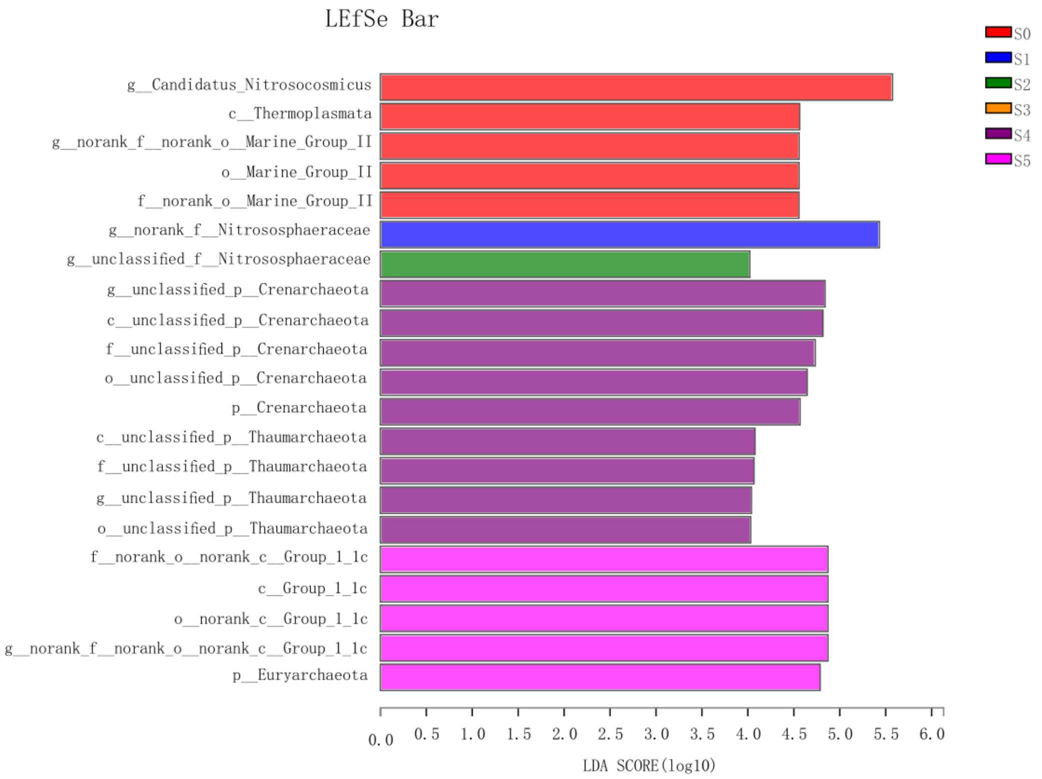

Supplementary Figure 7. LEfSe analysis. The histogram of the Linear discriminant analysis (LDA) scores illustrates the differentially abundant archaeal communities along successional stages.

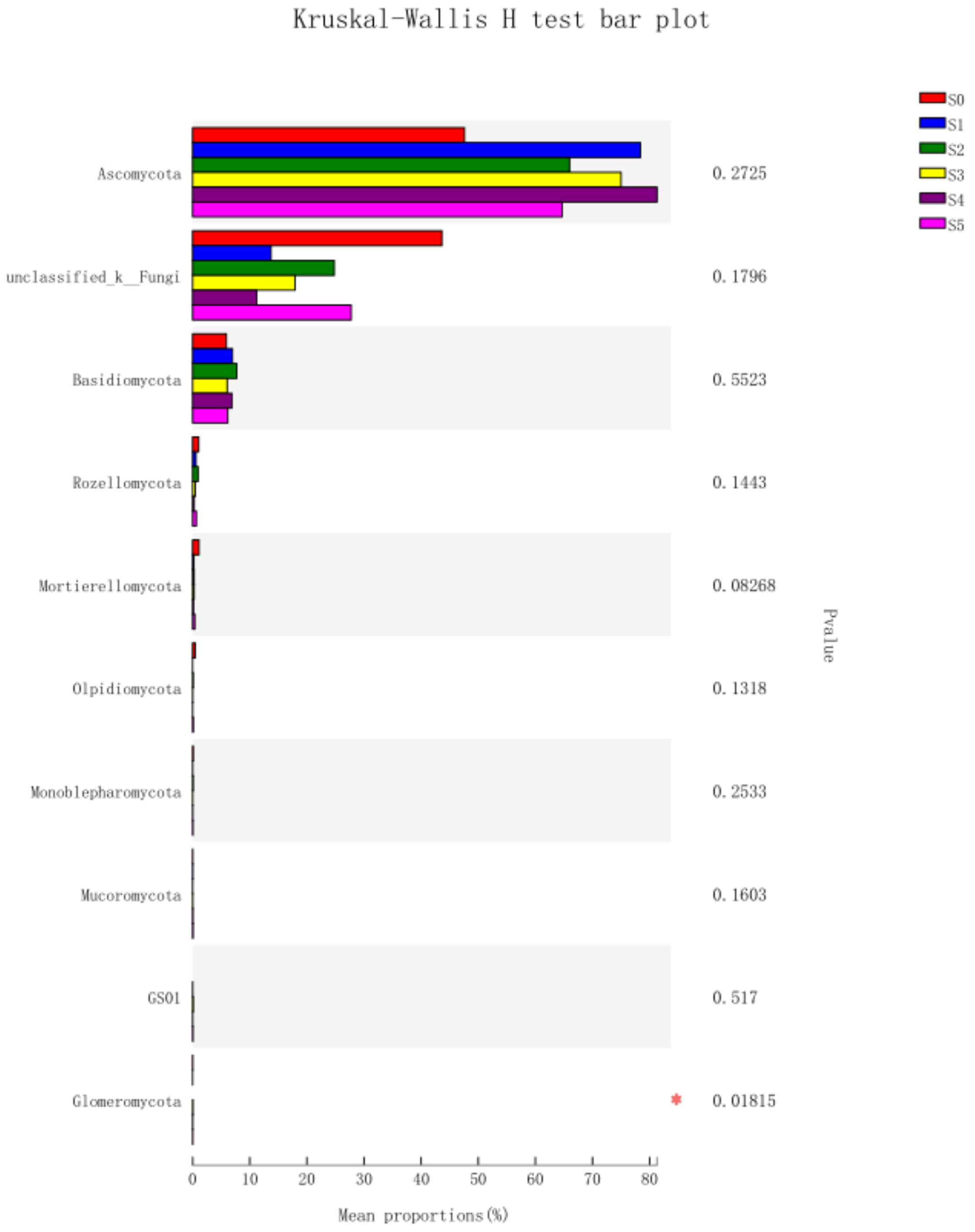

Supplementary Figure 8. Differential fungal phyla levels comparison between different successional stages. * 0.01 < P ≤ 0.05, ** 0.001 < P ≤ 0.01, and *** P ≤ 0.001.

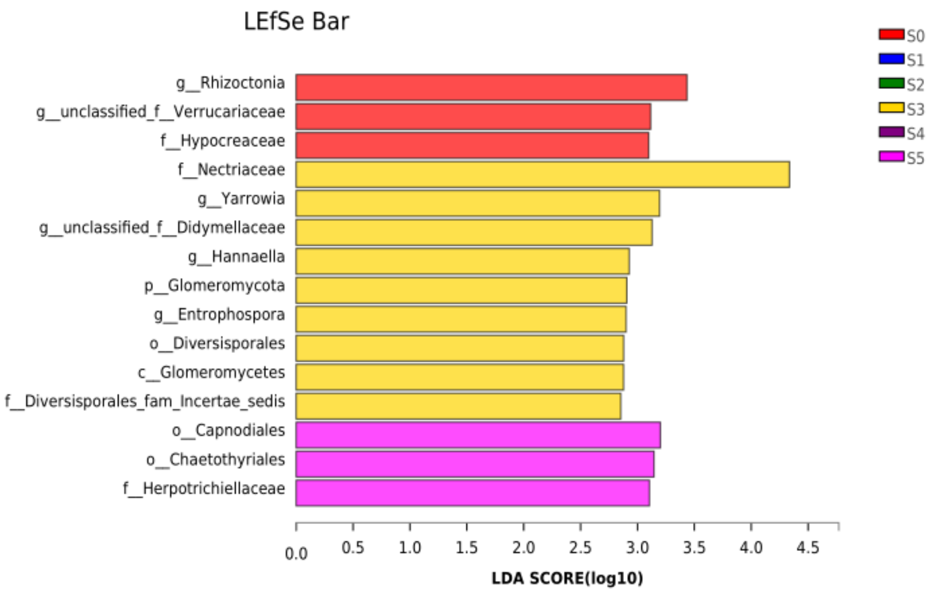

Supplementary Figure 9. LEfSe analysis. The histogram of the Linear discriminant analysis (LDA) scores illustrates the differentially abundant fungal communities along successional stages.
