# Supplementary material for: Antarctic Ardley Island terrace — An ideal place to study the marine to terrestrial succession of microbial communities
Source: Front Microbiol. 2023 Feb 6;14:942428. doi: 10.3389/fmicb.2023.942428 (PMC9940900; doi:10.3389/fmicb.2023.942428)
Supplement: Supplementary file 3 [file Table_1.DOCX]

Supplementary Material

Table

**Supplementary Table S1.** Soil elemental compositions investigated in this study.

| **Factors** | **Al** | **Br** | **Ca** | **Cl** | **Cr** | **Cu** | **Fe** | **K** | **Mg** | **Mn** | **Na** | **P** | **S** | **Si** | **Sr** | **Ti** | **Zn** | **Zr** |
| --- | --- | --- | --- | --- | --- | --- | --- | --- | --- | --- | --- | --- | --- | --- | --- | --- | --- | --- |
| **S0-0** | 9.636 | 0.005 | 3.454 | 0.030 | 0.066 | 0.014 | 5.919 | 1.110 | 2.248 | 0.116 | 2.750 | 0.202 | 0.060 | 26.380 | 0.051 | 0.562 | 0.012 | 0.015 |
| **S0-1** | 9.840 | 0.000 | 3.042 | 0.016 | 0.054 | 0.012 | 5.729 | 1.180 | 2.205 | 0.118 | 2.750 | 0.192 | 0.052 | 27.090 | 0.053 | 0.480 | 0.011 | 0.014 |
| **S0-2** | 9.389 | 0.000 | 3.335 | 0.036 | 0.051 | 0.011 | 5.977 | 1.100 | 2.075 | 0.116 | 2.740 | 0.298 | 0.086 | 25.850 | 0.049 | 0.592 | 0.007 | 0.016 |
| **S0-3** | 9.386 | 0.015 | 3.040 | 0.035 | 0.051 | 0.010 | 5.640 | 1.120 | 2.092 | 0.124 | 2.650 | 0.259 | 0.105 | 25.480 | 0.051 | 0.506 | 0.009 | 0.014 |
| **S0-4** | 9.755 | 0.000 | 3.147 | 0.024 | 0.066 | 0.015 | 5.984 | 1.180 | 2.199 | 0.132 | 2.760 | 0.192 | 0.039 | 27.090 | 0.051 | 0.554 | 0.009 | 0.015 |
| **S1-0** | 7.397 | 0.031 | 3.760 | 0.132 | 0.023 | 0.010 | 6.196 | 0.468 | 1.406 | 0.110 | 2.000 | 0.575 | 0.248 | 16.600 | 0.039 | 0.849 | 0.007 | 0.015 |
| **S1-1** | 6.714 | 0.046 | 2.992 | 0.182 | 0.022 | 0.010 | 5.607 | 0.478 | 1.180 | 0.088 | 1.830 | 0.662 | 0.340 | 14.850 | 0.043 | 0.739 | 0.007 | 0.014 |
| **S1-2** | 7.991 | 0.033 | 3.790 | 0.110 | 0.040 | 0.013 | 6.352 | 0.635 | 1.590 | 0.110 | 2.290 | 0.547 | 0.219 | 18.920 | 0.048 | 0.786 | 0.008 | 0.015 |
| **S1-3** | 7.177 | 0.044 | 3.019 | 0.166 | 0.025 | 0.011 | 5.990 | 0.477 | 1.160 | 0.093 | 1.810 | 0.963 | 0.366 | 14.930 | 0.042 | 0.817 | 0.007 | 0.015 |
| **S1-4** | 8.467 | 0.018 | 4.479 | 0.092 | 0.037 | 0.010 | 6.808 | 0.603 | 1.724 | 0.113 | 2.500 | 0.343 | 0.163 | 20.580 | 0.051 | 0.927 | 0.009 | 0.017 |
| **S2-0** | 7.126 | 0.037 | 4.118 | 0.134 | 0.019 | 0.013 | 5.611 | 0.580 | 0.856 | 0.081 | 1.820 | 2.620 | 0.334 | 14.070 | 0.052 | 0.801 | 0.010 | 0.015 |
| **S2-1** | 6.063 | 0.044 | 3.418 | 0.160 | 0.014 | 0.008 | 5.555 | 0.335 | 1.090 | 0.076 | 1.680 | 0.471 | 0.395 | 13.590 | 0.032 | 0.836 | 0.006 | 0.015 |
| **S2-2** | 6.915 | 0.045 | 3.778 | 0.166 | 0.000 | 0.009 | 5.851 | 0.387 | 1.290 | 0.091 | 2.060 | 0.623 | 0.286 | 15.440 | 0.034 | 0.864 | 0.006 | 0.016 |
| **S2-3** | 7.747 | 0.029 | 3.823 | 0.110 | 0.045 | 0.010 | 6.196 | 0.613 | 1.190 | 0.083 | 2.320 | 1.260 | 0.225 | 18.180 | 0.043 | 0.801 | 0.006 | 0.016 |
| **S2-4** | 6.664 | 0.058 | 3.366 | 0.167 | 0.022 | 0.010 | 6.172 | 0.342 | 1.150 | 0.075 | 1.870 | 0.654 | 0.372 | 14.240 | 0.034 | 0.882 | 0.006 | 0.016 |
| **S3-0** | 7.831 | 0.033 | 5.817 | 0.097 | 0.023 | 0.017 | 6.602 | 0.626 | 0.726 | 0.100 | 1.820 | 4.643 | 0.230 | 13.930 | 0.078 | 0.916 | 0.017 | 0.017 |
| **S3-1** | 6.907 | 0.037 | 3.037 | 0.143 | 0.017 | 0.009 | 5.721 | 0.527 | 1.070 | 0.067 | 1.870 | 1.330 | 0.366 | 15.120 | 0.040 | 0.732 | 0.006 | 0.015 |
| **S3-2** | 7.291 | 0.046 | 4.127 | 0.125 | 0.021 | 0.011 | 7.109 | 0.511 | 1.220 | 0.100 | 2.160 | 0.801 | 0.255 | 16.950 | 0.045 | 0.949 | 0.008 | 0.017 |
| **S3-3** | 7.762 | 0.020 | 3.898 | 0.110 | 0.031 | 0.009 | 6.267 | 0.604 | 1.434 | 0.083 | 2.320 | 0.807 | 0.209 | 18.970 | 0.042 | 0.799 | 0.007 | 0.016 |
| **S3-4** | 6.439 | 0.036 | 3.448 | 0.127 | 0.000 | 0.008 | 5.914 | 0.332 | 1.100 | 0.081 | 1.690 | 0.716 | 0.380 | 13.480 | 0.034 | 0.875 | 0.007 | 0.015 |
| **S4-0** | 6.203 | 0.034 | 3.405 | 0.111 | 0.000 | 0.008 | 5.712 | 0.320 | 1.080 | 0.086 | 1.690 | 0.488 | 0.330 | 13.360 | 0.031 | 0.810 | 0.005 | 0.015 |
| **S4-1** | 6.287 | 0.057 | 3.495 | 0.146 | 0.000 | 0.007 | 5.822 | 0.329 | 1.150 | 0.082 | 1.800 | 0.435 | 0.299 | 14.130 | 0.030 | 0.869 | 0.006 | 0.015 |
| **S4-2** | 7.425 | 0.033 | 5.783 | 0.099 | 0.023 | 0.018 | 6.571 | 0.748 | 0.729 | 0.082 | 1.720 | 4.807 | 0.250 | 13.450 | 0.071 | 0.847 | 0.017 | 0.017 |
| **S4-3** | 7.208 | 0.019 | 4.073 | 0.108 | 0.027 | 0.008 | 6.303 | 0.418 | 1.369 | 0.096 | 2.350 | 0.453 | 0.244 | 17.090 | 0.038 | 0.902 | 0.007 | 0.017 |
| **S4-4** | 7.093 | 0.048 | 3.105 | 0.142 | 0.000 | 0.012 | 6.188 | 0.402 | 0.939 | 0.070 | 1.710 | 2.090 | 0.344 | 13.290 | 0.040 | 0.781 | 0.006 | 0.015 |
| **S5-0** | 7.082 | 0.058 | 2.755 | 0.156 | 0.019 | 0.015 | 6.111 | 0.565 | 0.791 | 0.065 | 1.600 | 2.290 | 0.371 | 12.820 | 0.036 | 0.867 | 0.007 | 0.016 |
| **S5-1** | 6.199 | 0.081 | 2.308 | 0.225 | 0.014 | 0.013 | 5.440 | 0.395 | 0.719 | 0.053 | 1.430 | 2.140 | 0.484 | 11.180 | 0.029 | 0.766 | 0.006 | 0.014 |
| **S5-2** | 7.238 | 0.043 | 3.047 | 0.129 | 0.028 | 0.011 | 5.725 | 0.743 | 0.820 | 0.067 | 1.910 | 2.470 | 0.326 | 15.250 | 0.040 | 0.742 | 0.008 | 0.015 |
| **S5-3** | 6.525 | 0.054 | 2.560 | 0.147 | 0.019 | 0.012 | 5.596 | 0.389 | 0.698 | 0.073 | 1.480 | 1.840 | 0.411 | 11.950 | 0.033 | 0.821 | 0.005 | 0.015 |
| **S5-4** | 6.980 | 0.048 | 3.081 | 0.127 | 0.016 | 0.016 | 6.102 | 0.590 | 0.692 | 0.070 | 1.670 | 3.030 | 0.368 | 13.230 | 0.041 | 0.815 | 0.007 | 0.016 |

**Supplementary Table S2.** Distribution of bacteria, archaea and fungi in Ardley Island coastal uplifted chronosequence at each classification level

# Distribution of bacterial species at different classification levels

| **Sample** | **Phylum** | **Class** | **Order** | **Family** | **Genus** | **Species** |
| --- | --- | --- | --- | --- | --- | --- |
| S0-0 | 23 | 66 | 95 | 92 | 61 | 14 |
| S0-1 | 29 | 79 | 112 | 107 | 82 | 14 |
| S0-2 | 27 | 76 | 106 | 101 | 77 | 17 |
| S0-3 | 27 | 81 | 109 | 106 | 71 | 13 |
| S0-4 | 29 | 81 | 110 | 105 | 75 | 14 |
| S1-0 | 29 | 79 | 104 | 94 | 67 | 14 |
| S1-1 | 25 | 69 | 98 | 92 | 75 | 23 |
| S1-2 | 27 | 75 | 103 | 95 | 57 | 16 |
| S1-3 | 24 | 72 | 93 | 90 | 62 | 14 |
| S1-4 | 23 | 63 | 84 | 70 | 38 | 11 |
| S2-0 | 26 | 72 | 90 | 77 | 57 | 14 |
| S2-1 | 29 | 78 | 100 | 78 | 44 | 8 |
| S2-2 | 25 | 69 | 92 | 74 | 46 | 10 |
| S2-3 | 29 | 81 | 103 | 84 | 62 | 12 |
| S2-4 | 27 | 80 | 96 | 78 | 53 | 13 |
| S3-0 | 28 | 72 | 92 | 85 | 60 | 20 |
| S3-1 | 29 | 77 | 97 | 81 | 51 | 18 |
| S3-2 | 27 | 73 | 88 | 66 | 37 | 5 |
| S3-3 | 27 | 75 | 100 | 91 | 75 | 22 |
| S3-4 | 24 | 72 | 93 | 75 | 49 | 12 |
| S4-0 | 30 | 79 | 100 | 86 | 57 | 13 |
| S4-1 | 27 | 77 | 104 | 93 | 69 | 24 |
| S4-2 | 28 | 73 | 91 | 87 | 58 | 18 |
| S4-3 | 29 | 77 | 108 | 93 | 66 | 17 |
| S4-4 | 27 | 76 | 96 | 87 | 61 | 15 |
| S5-0 | 26 | 68 | 78 | 72 | 51 | 13 |
| S5-1 | 25 | 68 | 84 | 78 | 52 | 13 |
| S5-2 | 28 | 73 | 90 | 86 | 63 | 18 |
| S5-3 | 24 | 73 | 94 | 90 | 70 | 21 |
| S5-4 | 25 | 72 | 88 | 78 | 52 | 17 |

# Distribution of archaeal species at different classification levels

| **Sample** | **Phylum** | **Class** | **Order** | **Family** | **Genus** | **Species** |
| --- | --- | --- | --- | --- | --- | --- |
| S0-0 | 2 | 4 | 3 | 6 | 3 | 3 |
| S0-1 | 2 | 4 | 3 | 6 | 3 | 3 |
| S0-2 | 2 | 4 | 3 | 6 | 3 | 3 |
| S0-3 | 2 | 4 | 3 | 6 | 3 | 2 |
| S0-4 | 2 | 4 | 3 | 5 | 2 | 2 |
| S1-0 | 2 | 3 | 3 | 2 | 2 | 2 |
| S1-1 | 2 | 4 | 4 | 3 | 2 | 2 |
| S1-2 | 2 | 2 | 2 | 2 | 2 | 2 |
| S1-3 | 2 | 3 | 4 | 2 | 2 | 2 |
| S1-4 | 2 | 4 | 4 | 3 | 2 | 3 |
| S2-0 | 2 | 3 | 4 | 2 | 1 | 1 |
| S2-1 | 1 | 2 | 3 | 1 | 1 | 2 |
| S2-2 | 1 | 2 | 3 | 1 | 1 | 2 |
| S2-3 | 2 | 3 | 3 | 1 | 1 | 2 |
| S2-4 | 1 | 2 | 3 | 1 | 1 | 2 |
| S3-0 | 2 | 3 | 3 | 2 | 1 | 1 |
| S3-1 | 2 | 3 | 4 | 1 | 1 | 2 |
| S3-2 | 1 | 2 | 2 | 1 | 1 | 2 |
| S3-3 | 2 | 3 | 4 | 2 | 2 | 1 |
| S3-4 | 1 | 2 | 3 | 2 | 2 | 3 |
| S4-0 | 1 | 2 | 3 | 1 | 1 | 2 |
| S4-1 | 3 | 4 | 5 | 2 | 1 | 3 |
| S4-2 | 2 | 3 | 4 | 2 | 2 | 2 |
| S4-3 | 3 | 4 | 5 | 2 | 2 | 2 |
| S4-4 | 2 | 4 | 6 | 4 | 3 | 2 |
| S5-0 | 1 | 2 | 4 | 2 | 2 | 2 |
| S5-1 | 1 | 2 | 3 | 2 | 2 | 3 |
| S5-2 | 1 | 2 | 4 | 2 | 2 | 1 |
| S5-3 | 3 | 5 | 7 | 5 | 4 | 2 |
| S5-4 | 1 | 2 | 4 | 2 | 2 | 3 |

**Supplementary Table S3.** Results of Adonis analysis (PERMANOVA) between environmental factors and microbial communities

# Bacterial communities

| **Characteristics** | **SumsOfSqs** | **MeanSqs** | **F.Model** | **R2** | **P.value** | **P.adjust** |
| --- | --- | --- | --- | --- | --- | --- |
| Mg | 1.28893 | 1.28893 | 14.00418 | 0.3334 | 0.001 | 0.0016 |
| Si | 1.26739 | 1.26739 | 13.65609 | 0.32783 | 0.001 | 0.0016 |
| Al | 1.13322 | 1.13322 | 11.61089 | 0.29312 | 0.001 | 0.0016 |
| Na | 1.10548 | 1.10548 | 11.21279 | 0.28595 | 0.001 | 0.0016 |
| K | 1.08936 | 1.08936 | 10.98525 | 0.28178 | 0.001 | 0.0016 |
| S | 1.07385 | 1.07385 | 10.76868 | 0.27777 | 0.001 | 0.0016 |
| Ti | 1.06151 | 1.06151 | 10.59798 | 0.27457 | 0.001 | 0.0016 |
| pH | 1.04429 | 1.04429 | 10.36249 | 0.27012 | 0.001 | 0.0016 |
| Moisture | 1.03831 | 1.03831 | 10.28135 | 0.26857 | 0.001 | 0.0016 |
| Mn | 1.02512 | 1.02512 | 10.10359 | 0.26516 | 0.001 | 0.0016 |
| Cl | 1.00353 | 1.00353 | 9.81629 | 0.25958 | 0.001 | 0.0016 |
| Br | 0.97971 | 0.97971 | 9.50417 | 0.25342 | 0.001 | 0.0016 |
| Cr | 0.95336 | 0.95336 | 9.16489 | 0.2466 | 0.001 | 0.0016 |
| VC | 0.89537 | 0.89537 | 8.43937 | 0.2316 | 0.001 | 0.0016 |
| NH_4_ | 0.83926 | 0.83926 | 7.76381 | 0.21709 | 0.001 | 0.0016 |
| P | 0.62973 | 0.62973 | 5.44839 | 0.16289 | 0.002 | 0.00282 |
| TOC | 0.56421 | 0.56421 | 4.78459 | 0.14594 | 0.002 | 0.00282 |
| Cu | 0.4312 | 0.4312 | 3.51507 | 0.11154 | 0.014 | 0.01867 |
| Sr | 0.2482 | 0.2482 | 1.92098 | 0.0642 | 0.07 | 0.08842 |
| Zn | 0.19514 | 0.19514 | 1.48845 | 0.05048 | 0.147 | 0.1764 |
| Zr | 0.14356 | 0.14356 | 1.07988 | 0.03713 | 0.302 | 0.34514 |
| Ca | 0.1381 | 0.1381 | 1.03725 | 0.03572 | 0.369 | 0.40255 |
| Fe | 0.12546 | 0.12546 | 0.93914 | 0.03245 | 0.439 | 0.45809 |
| NO_3_ | 0.10661 | 0.10661 | 0.794 | 0.02758 | 0.572 | 0.572 |

# Archaeal communities

| **Characteristics** | **SumsOfSqs** | **MeanSqs** | **F.Model** | **R2** | **P.value** | **P.adjust** |
| --- | --- | --- | --- | --- | --- | --- |
| Mg | 2.52419 | 2.52419 | 20.37381 | **0.42117** | 0.001 | 0.0015 |
| Si | 2.26635 | 2.26635 | 17.02711 | 0.37815 | 0.001 | 0.0015 |
| Na | 2.16564 | 2.16564 | 15.84234 | 0.36135 | 0.001 | 0.0015 |
| Mn | 1.97694 | 1.97694 | 13.78249 | 0.32986 | 0.001 | 0.0015 |
| NH_4_ | 1.95706 | 1.95706 | 13.57674 | 0.32655 | 0.001 | 0.0015 |
| S | 1.89745 | 1.89745 | 12.97161 | 0.3166 | 0.001 | 0.0015 |
| Br | 1.7952 | 1.7952 | 11.97364 | 0.29954 | 0.001 | 0.0015 |
| Al | 1.64904 | 1.64904 | 10.62872 | 0.27515 | 0.001 | 0.0015 |
| Ti | 1.63514 | 1.63514 | 10.50558 | 0.27283 | 0.001 | 0.0015 |
| VC | 1.55462 | 1.55462 | 9.80705 | 0.2594 | 0.001 | 0.0015 |
| TOC | 1.49987 | 1.49987 | 9.34636 | 0.25026 | 0.001 | 0.0015 |
| Cr | 1.49232 | 1.49232 | 9.28369 | 0.249 | 0.001 | 0.0015 |
| Cl | 1.47461 | 1.47461 | 9.13753 | 0.24605 | 0.001 | 0.0015 |
| K | 1.4595 | 1.4595 | 9.01384 | 0.24353 | 0.001 | 0.0015 |
| Moisture | 1.39965 | 1.39965 | 8.53153 | 0.23354 | 0.002 | 0.00282 |
| P | 1.35419 | 1.35419 | 8.17352 | 0.22595 | 0.001 | 0.0015 |
| pH | 1.20999 | 1.20999 | 7.08302 | 0.20189 | 0.001 | 0.0015 |
| Cu | 0.25095 | 0.25095 | 1.22365 | 0.04187 | 0.287 | 0.38267 |
| Fe | 0.19899 | 0.19899 | 0.9616 | 0.0332 | 0.39 | 0.49263 |
| Sr | 0.18037 | 0.18037 | 0.86883 | 0.0301 | 0.44 | 0.528 |
| Ca | 0.1368 | 0.1368 | 0.65403 | 0.02283 | 0.575 | 0.65714 |
| Zr | 0.1286 | 0.1286 | 0.61399 | 0.02146 | 0.619 | 0.67527 |
| Zn | 0.11091 | 0.11091 | 0.52794 | 0.01851 | 0.721 | 0.75235 |
| NO_3_ | 0.08304 | 0.08304 | 0.39341 | 0.01386 | 0.87 | 0.87 |

# Fungal communities

| **Characteristics** | **SumsOfSqs** | **MeanSqs** | **F.Model** | **R2** | **P.value** | **P.adjust** |
| --- | --- | --- | --- | --- | --- | --- |
| Al | 0.57038 | 0.57038 | 3.36754 | 0.10736 | 0.024 | 0.21 |
| Ti | 0.56701 | 0.56701 | 3.34526 | 0.10672 | 0.024 | 0.21 |
| K | 0.55309 | 0.55309 | 3.25355 | 0.1041 | 0.035 | 0.21 |
| Cl | 0.49021 | 0.49021 | 2.84605 | 0.09227 | 0.043 | 0.21 |
| Cr | 0.45272 | 0.45272 | 2.60813 | 0.08521 | 0.053 | 0.21 |
| Si | 0.43841 | 0.43841 | 2.51827 | 0.08252 | 0.06 | 0.21 |
| S | 0.42154 | 0.42154 | 2.41306 | 0.07934 | 0.07 | 0.21 |
| NH_4_ | 0.40004 | 0.40004 | 2.27996 | 0.0753 | 0.063 | 0.21 |
| NO_3_ | 0.33794 | 0.33794 | 1.90195 | 0.06361 | 0.127 | 0.25543 |
| Mn | 0.33783 | 0.33783 | 1.90129 | 0.06359 | 0.115 | 0.25543 |
| pH | 0.32919 | 0.32919 | 1.84947 | 0.06196 | 0.122 | 0.25543 |
| Na | 0.32795 | 0.32795 | 1.84202 | 0.06173 | 0.142 | 0.25543 |
| Mg | 0.31211 | 0.31211 | 1.74755 | 0.05875 | 0.146 | 0.25543 |
| Moisture | 0.29904 | 0.29904 | 1.66997 | 0.05628 | 0.149 | 0.25543 |
| Br | 0.28995 | 0.28995 | 1.61631 | 0.05457 | 0.181 | 0.2896 |
| TOC | 0.25156 | 0.25156 | 1.39166 | 0.04735 | 0.194 | 0.291 |
| VC | 0.24778 | 0.24778 | 1.36973 | 0.04664 | 0.212 | 0.29929 |
| Zn | 0.22465 | 0.22465 | 1.23621 | 0.04228 | 0.273 | 0.3276 |
| Sr | 0.22321 | 0.22321 | 1.22791 | 0.04201 | 0.265 | 0.3276 |
| Cu | 0.21984 | 0.21984 | 1.20859 | 0.04138 | 0.251 | 0.3276 |
| Fe | 0.14783 | 0.14783 | 0.80141 | 0.02783 | 0.491 | 0.56114 |
| P | 0.13071 | 0.13071 | 0.70624 | 0.0246 | 0.53 | 0.57818 |
| Zr | 0.08986 | 0.08986 | 0.48175 | 0.01691 | 0.752 | 0.7847 |
| Ca | 0.07039 | 0.07039 | 0.37596 | 0.01325 | 0.864 | 0.864 |

*The first column of the table represents environmental factors; R2 value represents the interpretation degree of environmental factors to sample differences; the greater R2 means the higher the interpretation degree of environmental factors to sample differences; environmental factors were considered to significantly contribute to community variation at P<0.05*
